# The effect of postoperative rehabilitation on outcomes in patients with degenerative cervical myelopathy (DCM): A systematic review

**DOI:** 10.1016/j.bas.2026.105956

**Published:** 2026-02-02

**Authors:** Chanelle Montpetit, Adam Kobaisi, Justin M. Lantz, Rohil V. Chauhan, David B. Anderson, Maryse Fortin

**Affiliations:** a Department of Health, Kinesiology and Applied Physiology, Concordia University, Montreal, QC, Canada; b School of Health, Concordia University, Montreal, QC, Canada; c Centre de réadaptation Constance-Lethbridge du CIUSS COMTL, Montreal, QC, Canada; d USC Spine Physical Therapy Fellowship Program, Division of Biokinesiology and Physical Therapy, University of Southern California, Los Angeles, CA, USA; e Department of Orthopaedic Physiotherapy, Auckland Spine Surgery Centre, Auckland, New Zealand; f Active Living and Rehabilitation: Aotearoa New Zealand, Health and Rehabilitation Research Institute, Faculty of Health and Environmental Sciences, Auckland University of Technology, Auckland, New Zealand; g School of Health Sciences, Faculty of Medicine and Health, The University of Sydney, Camperdown, NSW, Australia

**Keywords:** Degenerative cervical myelopathy, Systematic review, Postoperative, Rehabilitation

## Abstract

**Introduction:**

Degenerative cervical myelopathy (DCM) is the leading cause of adult spinal cord dysfunction, often requiring surgery. However, the role of postoperative rehabilitation in optimizing patient outcomes remains unclear.

**Research question:**

What are the effects of postoperative rehabilitation on clinical outcomes following DCM surgery?

**Material and methods:**

This systematic review was registered with PROSPERO (CRD42024582484). PubMed, Scopus, and Web of Science were searched through September 2025. Eligible studies included randomized controlled trials (RCTs), and other research on rehabilitation interventions for postoperative outcomes (e.g., function, pain, neurological recovery) in patients undergoing DCM surgery. Studies without confirmed DCM, non-peer-reviewed articles, or lacking a rehabilitation protocol were excluded. Risk of bias was assessed using the RoB 2 and ROBINS-I. Descriptive summaries were conducted, categorizing studies into active, passive, and mixed interventions. The evidence quality was rated using the GRADE approach.

**Results:**

Ten studies with a total of 766 patients were included, made up of 5 RCTs and 5 cohort studies. Seven studies had high risk of bias, and three had moderate risk of bias. Mixed rehabilitation interventions combining physical, behavioral, and psychosocial strategies yielded the most consistent improvements in neurological function, quality of life, and self-efficacy. Intervention timing ranged from a few days postoperatively to 6 months. A meta-analysis was not performed due to study heterogeneity.

**Discussion and Conclusion:**

Postoperative rehabilitation for DCM shows promise, particularly with multimodal, goal-oriented, and patient-centered approaches. However, evidence is limited by the high risk of bias, poor methodological detail and lack of standardization.

## Highlights

• DCM postoperative rehabilitation effects were systematically reviewed.
• Mixed, multimodal interventions showed the most consistent clinical benefits.
• Improvements seen in neurological function, quality of life, and self-efficacy.
• Evidence quality was limited by high risk of bias and study heterogeneity.
• Standardized, high-quality RCTs are needed to guide postoperative DCM care.

## 1 Introduction

Degenerative cervical myelopathy (DCM) is the leading cause of spinal cord dysfunction worldwide, affecting approximately 2.3% of the adult population (Chauhan et al., 2025; Agrawal et al., 2025). DCM is marked by progressive degeneration of the cervical spine, leading to narrowing of the spinal canal and subsequent spinal cord compression (Thompson et al., 2024). While most common in older adults due to age-related changes such as disc dehydration, osteophyte formation, other risk factors include congenital spinal canal narrowing, movement disorders, and lifestyle factors (Thompson et al., 2024; Baucher et al., 2022). DCM may develop from structural and functional disruptions of the spinal cord, resulting from static compression (e.g., spondylotic changes) or dynamic forces (e.g., repetitive microtrauma), leading to cellular damage, ischemia, and secondary cellular responses such as inflammation and apoptosis (Choi and Kang, 2020). Clinically, DCM presents with a wide spectrum of symptoms, from pain, impaired balance, reduced hand dexterity and gait disturbances to paralysis, bladder dysfunction, and increased fall risk (Thompson et al., 2024)^,4,5^. These impairments can significantly reduce overall quality of life, interfering with daily activities and occupational capabilities (Choi and Kang, 2020).

Surgery is the recommended treatment for moderate, severe and progressive DCM, aiming to decompress the spinal cord and prevent further neurological decline (Kato and Fehlings, 2016). While advances have been made in surgical approach selection and indications, the extent of postoperative recovery of longstanding neurological impairment varies greatly (Choi and Kang, 2020; Kato and Fehlings, 2016). Many patients experience only partial recovery, and improvements may plateau over time, highlighting the complexities in predicting surgical success (Cloney et al., 2018; Fehlings et al., 2017). Delays in DCM diagnosis and subsequent decompressive surgery contribute to chronic impairments, which often persist despite technically successful surgery (Johansen et al., 2024).

Postoperative rehabilitation plays a critical role in recovery following spine surgical interventions (Barbosa et al., 2023; Lantz et al., 2025). Existing protocols for postoperative spine rehabilitation are varied and often include structured physical therapy aimed at improving functional recovery. However, due to its unique pathophysiology, DCM may require a rehabilitation approach that differs from that of other spine injuries (Rahman et al., 2024). Patient-centered rehabilitation that targets neurological recovery through fine motor training, gait retraining, and balance exercises may be more effective for individuals with DCM than standard care, yet evidence specific to DCM is limited (Chauhan et al., 2025; Lantz et al., 2021; Ling et al., 2023; Badran et al., 2018). Most existing reviews focus on broader cervical conditions, leaving a gap in evidence-based guidelines for postoperative rehabilitation in DCM (Chauhan et al., 2025).

With the emergence of new studies, the opportunity now exists to address these gaps by integrating updated evidence and evaluating both subjective and objective outcome measures, thereby providing a more thorough understanding of the impact of rehabilitation on this population. Therefore, the objective of this systematic review was to assess the effect of postoperative rehabilitation on clinical outcomes, including pain, function, quality of life, muscle strength, range of motion, and overall neurological improvement in patients with DCM, ranging from right after surgery to 12 months post-surgery.

## 2 Methods

This systematic review protocol was registered with PROSPERO (CRD42024582484) and conducted in accordance with the Preferred Reporting Items for Systematic Reviews and Meta-Analyses (PRISMA) 2020 guidelines (Page et al., 2021).

### 2.1 Selection criteria

Studies were included if they assessed postoperative rehabilitation in adults following surgery for a confirmed diagnosis of DCM. Eligible study designs included randomized and non-randomized controlled trials, longitudinal studies, and cohort studies, with a minimum of 8 participants per group. Only peer-reviewed English or French articles with clear methodologies and sufficient outcome data were considered. Refer to Table 1 for detailed inclusion and exclusion criteria.

### 2.2 Search strategy

Key search terms relating to three primary concepts: 1) degenerative cervical myelopathy (DCM), 2) postoperative, and 3) rehabilitation, were developed in consultation with a university librarian. The search strategy incorporated both free-text terms and controlled vocabulary (e.g., MeSH terms) to improve accuracy and sensitivity. The search terms were developed using synonyms and variations of key concepts related to the population, intervention, and outcomes.

Searches were conducted across PubMed (via the National Library of Medicine), Scopus (via Elsevier), and Web of Science (via Clarivate Analytics), including all records up to September 2025. Filters were applied for language (English and French) and species (Humans). Full details of the final search strategy are provided in the Supplemental Material (Table S1).

### 2.3 Study selection

**Fig. 1:**
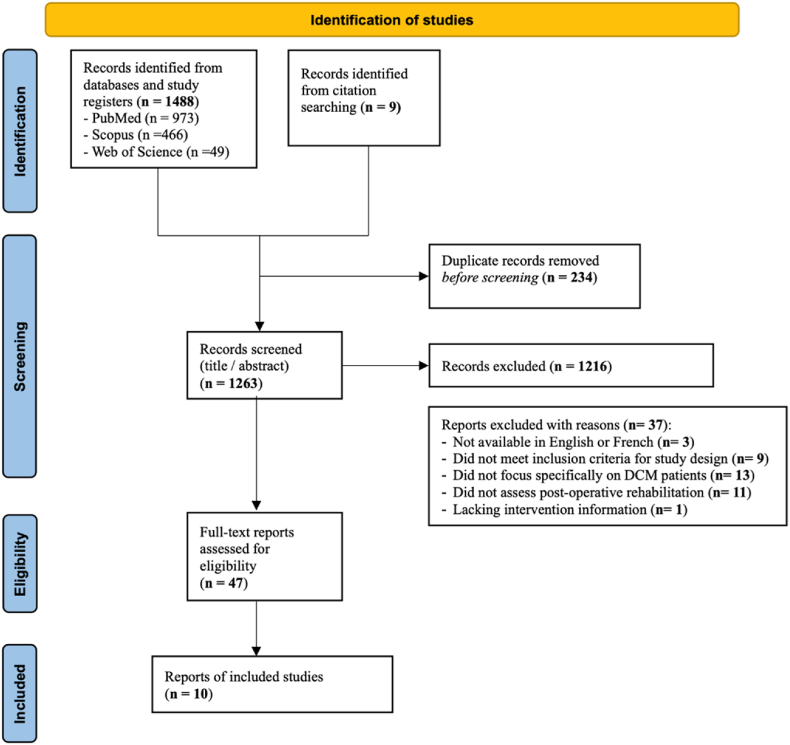
PRISMA flow diagram of study selection process.

Two authors (CM, AK) independently reviewed and screened titles and abstracts for potentially eligible studies, with clearly ineligible papers excluded and duplicates removed (Fig. 1). Two independent authors (CM, AK) assessed the full text of the remaining articles and a global yes/no decision was made for each study based on the predetermined eligibility criteria. In the case of disagreement over the eligibility of an article, a third reviewer (MF) was consulted, and a consensus decision between the 3 reviewers was taken. Study screening was managed using Rayyan.

### 2.4 Data extraction and risk of bias

Two authors (CM, AK) independently extracted data from each included study using a standardized Microsoft Excel spreadsheet. Extracted information focused on the following: author(s), year of publication, study design, population, sex, age, sample size, study arms (i.e., interventions), treatment details, treatment duration, outcome measures, time points assessed and key findings. Discrepancies were resolved through discussion, and if necessary, a third reviewer (MF) was consulted.

Outcome variables were extracted and categorized as follows:

● Patient-Reported Outcome Measures: pain (Visual Analog Scale, EQ-5D-5L), quality of life (EQ-5D-5L, SF-36, Japanese Orthopedic Association Cervical Myelopathy Evaluation Questionnaire [JOACMEQ]), disability (Neck Disability Index [NDI], JOACMEQ), self-efficacy (Self-Efficacy Rehabilitation scale [SER])
● Cervical spine impairments: neurological function (Modified Japanese Orthopedic Association [mJOA], Japanese Orthopedic Association [JOA]), cervical muscle strength, cervical range of motion, cervical spine alignment, motor function (American Spinal Injury Association [ASIA] motor scale), muscle tone (Modified Ashworth Scale)
● Functional outcomes: Barthel Index, grip strength, Timed Up and Go, Nurick Grade Scale
● Postoperative complications

Risk of bias was evaluated using two tools based on study design. For randomized trials, the Revised Cochrane Risk-of-Bias tool (RoB 2) was used (Sterne et al., 2019). This tool assesses bias across five domains: randomization process, deviations from intended interventions, missing outcome data, measurement of the outcome, and selection of the reported result. Each domain is rated as low risk, some concerns, or high risk using signaling questions and a structured algorithm. For non-randomized studies, the Risk Of Bias In Non-randomized Studies - of Interventions (ROBINS-I) tool was used (Sterne et al., 2016). This tool evaluates seven domains of bias: confounding, selection of participants, classification of interventions, deviations from intended interventions, missing data, measurement of outcomes, and selection of the reported result. The ROBINS-I tool guides reviewers to assess bias at three levels: low risk, moderate risk, and serious risk of bias using a structured framework.

Both reviewers adhered strictly to the guidance documents for each tool. In instances of disagreement, consensus was achieved through discussion or consultation with a third reviewer (MF).

### 2.5 Data analysis and reporting

Due to the heterogeneity of outcome measures and follow-up time points among included studies, a meta-analysis was not feasible. Thus, the data were synthesized using a descriptive summary, and the certainty of the overall body of evidence was assessed using the GRADE approach, following the guidelines outlined by the Cochrane Back and Neck Review Group (Furlan et al., 2015). The studies were sub-categorized into active (e.g., physical therapy), passive (e.g., cervical collar), and mixed interventions (e.g., exercise therapy combined with behavioral or educational components).

## 3 Results

### 3.1 Search results

The search yielded a total of 1497 records, reducing to 1263 following duplicate removal (Fig. 1). The full texts of 47 studies were assessed for eligibility, of which 10 were included in the final analysis. The main reasons for article exclusion were the inclusion of non-DCM-related participants and the lack of postoperative outcome measurement (Fig. 1).

### 3.2 Characteristics of included studies

This systematic review included ten studies evaluating postoperative rehabilitation for patients undergoing surgery for DCM, comprising five randomized controlled trials (Uehara et al., 2022; Lin et al., 2024; Farrokhi et al., 2024; Cheung et al., 2019; Yue and Liu, 2021), three prospective cohort studies (Rahman et al., 2024; Tamai et al., 2023; Cheng et al., 2020), and two retrospective cohort studies (Catz et al., 2024; Iizuka et al., 2005) (Table S2). Surgical procedures included anterior and posterior decompression, laminoplasty, and cervical fusion.

Rehabilitation interventions were diverse, with six studies using mixed rehabilitation approaches combining physical therapy, occupational therapy, or multidisciplinary programs with passive components (Rahman et al., 2024; Uehara et al., 2022; Lin et al., 2024; Farrokhi et al., 2024; Tamai et al., 2023; Catz et al., 2024) (Table S3). The mixed interventions typically included cervical stabilization exercises, range of motion training, gait training, muscle strengthening, and psychological support. Two studies focused on passive interventions (i.e., cervical collar use) (Cheung et al., 2019; Iizuka et al., 2005). One study investigated perturbation-based treadmill training (Cheng et al., 2020), and one evaluated repetitive transcranial magnetic stimulation (rTMS) combined with physiotherapy (Farrokhi et al., 2024).

Intervention durations ranged from 8 days to over a year, with assessments conducted at preoperative, short-term (weeks to months), and long-term (up to one year) follow-ups. The length of follow-up varied, with six studies evaluating short-term recovery (≤3 months) and four studies extending follow-up to one year or beyond. Study characteristics are provided in Table S2–S4.

Patient-reported outcome measures included pain (VAS; EQ-5D-5L pain domain) (Uehara et al., 2022; Cheung et al., 2019; Yue and Liu, 2021; Tamai et al., 2023; Cheng et al., 2020), quality of life (SF-36; EQ-5D-5L quality of life domain, JOACMEQ) (Rahman et al., 2024; Cheung et al., 2019; Yue and Liu, 2021; Tamai et al., 2023), disability (NDI; JOACMEQ) (Rahman et al., 2024; Uehara et al., 2022; Cheung et al., 2019; Tamai et al., 2023; Cheng et al., 2020), and self-efficacy (SER) (Lin et al., 2024). Cervical spine impairment outcomes included neurological function (modified Japanese Orthopedic Association [mJOA], Japanese Orthopedic Association [JOA] score) (Rahman et al., 2024; Lin et al., 2024; Farrokhi et al., 2024; Cheung et al., 2019; Yue and Liu, 2021; Tamai et al., 2023; Iizuka et al., 2005), cervical muscle strength (Uehara et al., 2022), cervical range of motion (Cheung et al., 2019; Yue and Liu, 2021; Iizuka et al., 2005), spine alignment (Uehara et al., 2022), motor function (ASIA scale) (Farrokhi et al., 2024; Catz et al., 2024), and muscle tone (Modified Ashworth Scale) (Farrokhi et al., 2024). Functional outcomes included the Barthel Index, Grip Strength, Timed Up and Go, and Nurick grade (Lin et al., 2024; Farrokhi et al., 2024; Cheng et al., 2020). Postoperative complications were also analyzed (Rahman et al., 2024; Cheung et al., 2019; Yue and Liu, 2021).

### 3.3 Risk of bias

**Fig. 2:**
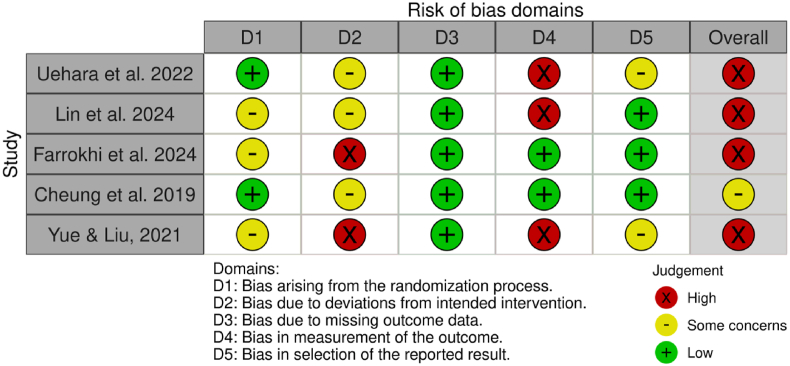
Risk of bias assessment for randomized controlled trials using the RoB 2 tool.

**Fig. 3:**
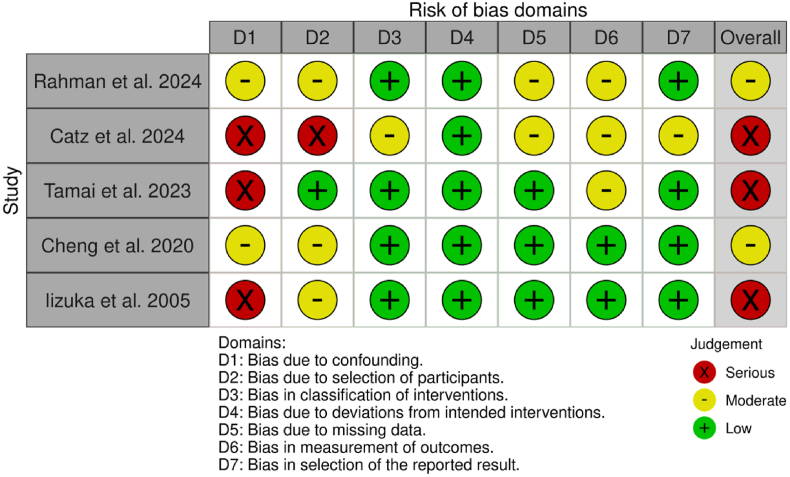
Risk of bias assessment for non-randomized studies using the ROBINS-I tool.

The included studies exhibited varying levels of bias, ranging from “moderate” to “severe” (Fig. 2, Fig. 3). Three studies were assessed as having a moderate risk of bias (Rahman et al., 2024; Cheung et al., 2019; Cheng et al., 2020), and seven studies were deemed to have a serious risk of bias (Uehara et al., 2022; Lin et al., 2024; Farrokhi et al., 2024; Yue and Liu, 2021; Tamai et al., 2023; Catz et al., 2024; Iizuka et al., 2005). No studies were classified as having a low risk of bias across all domains. Studies with a moderate risk of bias commonly faced issues such as confounding variables, selection bias, and potential deviations from intended interventions. In contrast, studies with a serious risk of bias had multiple methodological concerns, including inadequate control of baseline differences, selection bias from participant allocation, protocol deviations, and measurement bias due to lack of assessor blinding.

### 3.4 Patient-reported outcome measures

A complete summary of patient-reported outcome measures is presented in Tables S2 and S4. The primary outcome results for each study are presented in Table 2.

#### 3.4.1 Pain

Improvements in pain outcomes have been reported across five studies, using the Visual Analog Scale (VAS) scores and the pain subsection of EuroQoL 5-dimension 5-level (EQ-5D-5L) (Uehara et al., 2022; Cheung et al., 2019; Yue and Liu, 2021; Tamai et al., 2023; Cheng et al., 2020).

##### 3.4.1.1 Active interventions

Cheng et al. (2020) evaluated postoperative VAS pain outcomes in patients with DCM who underwent a 4-week perturbation-based balance training program compared to a healthy control group (no exercise). Although pain scores decreased slightly following the intervention, the change was not statistically significant (p = 1.00). Uehara et al. (2022) evaluated postoperative VAS axial pain in patients who received a cervical exercise program compared to those who did not. Both groups experienced an initial increase in pain two weeks after surgery, followed by a reduction at the three-month mark. While repeated-measures analysis showed a significant effect of time (p < 0.001), indicating an overall decrease in pain over the postoperative period, there were no significant between-group differences (p = 0.613).

##### 3.4.1.2 Passive interventions

Cheung et al. (2019) evaluated postoperative VAS axial neck pain in patients who wore a cervical collar compared to those who did not. Pain levels were similar between groups before surgery. While both groups experienced an increase in pain shortly after surgery, the cervical collar group reported lower pain levels during the early postoperative period. By one year postoperatively, pain levels had decreased in both groups and were comparable (p = 0.607).

##### 3.4.1.3 Mixed interventions

Tamai et al. (2023) evaluated postoperative VAS pain scores using a multidisciplinary rehabilitation approach compared to standard care. Both groups demonstrated non-significant reductions in neck pain, arm pain, and arm numbness at the one-year follow-up. Tamai et al. (2023) also evaluated postoperative pain using the EQ-5D-5L. Both groups demonstrated small improvements in pain scores one year after surgery, though the changes were not statistically significant (p = 0.117). Yue and Liu (2021) assessed postoperative VAS pain scores in patients who received Timeliness Incentive Nursing (TIN) compared to routine care. Both groups demonstrated significant reductions in pain following the intervention (p < 0.001), with the TIN group showing greater improvements than the routine care group (p < 0.001).

#### 3.4.2 Quality of life

Across four studies, improvements in both general and domain-specific quality of life scores were reported, albeit using varied outcome measures such as the Short Form 36 (SF-36) and EuroQoL 5-dimension 5-level (EQ-5D-5L), and JOACMEQ quality of life subsection (Rahman et al., 2024; Cheung et al., 2019; Yue and Liu, 2021; Tamai et al., 2023).

##### 3.4.2.1 Passive interventions

Cheung et al. (2019) evaluated postoperative care using a cervical collar vs. no cervical collar. They reported improvements in the SF-36 physical function and bodily pain. However, these changes did not reach statistical significance for the physical or mental component summary scores.

##### 3.4.2.2 Mixed interventions

Yue and Liu (2021) examined Timeliness Incentive Nursing (TIN), a model that integrated structured rehabilitation, motivational support, and timely clinical feedback, and reported statistically significant improvements in overall quality of life at two months post-intervention (p < 0.001). Using the SF-36, they found significant improvements in general health, with sustained improvements across multiple domains: physiological function, bodily pain, mental health, energy, social functioning, and physiological responsibility in both the TIN group and control group. Rahman et al. (2024) assessed a multimodal program, which incorporated both active components (goal setting, exercise) and passive components (education). They reported a statistically significant improvement in the SF-36 Physical Component Summary (PCS) score in the intervention group, particularly with occupational therapy (p = 0.009). Tamai et al. (2023) investigated a multidisciplinary approach (i.e., physical therapy, counselling, and medical care) compared to standard care. The EQ-5D-5L self-care domain improved significantly in the intervention group compared to the standard care group (p = 0.047). While other domains (pain, usual activities) showed non-significant trends favoring the multidisciplinary intervention, the total EQ-5D-5L score improvement did not reach statistical significance (p = 0.145). Quality of life, assessed also via a JOACMEQ subsection, improved from baseline to one year postoperatively in both groups. However, no significant between-groups differences were observed.

#### 3.4.3 Disability

Across five studies, improvements in disability were reported, with the main outcomes being the Neck Disability Index (NDI) and JOACMEQ (Rahman et al., 2024; Uehara et al., 2022; Cheung et al., 2019; Tamai et al., 2023; Cheng et al., 2020).

##### 3.4.3.1 Active interventions

Rahman et al. (2024) evaluated postoperative disability using the Neck Disability Index (NDI) in patients with DCM who underwent postsurgical rehabilitation that included both occupational therapy and physical therapy. Disability scores improved over the 12-month follow-up period; however, the study did not report whether this improvement was statistically significant. Cheng et al. (2020) evaluated postoperative disability using NDI in patients with DCM who underwent perturbation-based balance training. The study included a healthy control group, but this group did not receive standard postoperative care or any rehabilitation intervention. Disability scores improved slightly following the intervention; however, the change was not statistically significant (p = 0.30). Uehara et al. (2022) evaluated cervical spine function using the JOACMEQ questionnaire in patients who received postoperative cervical exercise versus those who received no exercise intervention. Both groups showed improvement at 3 months postoperatively, with slightly greater improvement in the exercise group compared to the control group; however, the difference was not significant (p = 0.52).

##### 3.4.3.2 Passive interventions

Cheung et al. (2019) assessed postoperative disability using the NDI in patients who wore a cervical collar compared to those who did not. Both groups began with similar disability scores preoperatively. At the one-year follow-up, both groups showed non-significant reductions in disability (p = 0.695), with slightly greater improvement observed in the cervical collar group.

##### 3.4.3.3 Mixed interventions

Tamai et al. (2023) assessed NDI scores, comparing patients who underwent a multidisciplinary rehabilitation program to those who received standard care. Both groups showed reductions in disability scores at the one-year follow-up. However, the changes were not statistically significant (p = 0.342). Cervical, upper extremity, and lower extremity function was also assessed using the JOACMEQ in patients who received a multidisciplinary approach compared to those who received standard care. Both groups demonstrated improvements across all domains at the one-year follow-up. Statistically significant improvement was observed in upper extremity function (p = 0.001), while changes in cervical and lower extremity function were not statistically significant (p = 0.118 and p = 0.060, respectively).

#### 3.4.4 Self-efficacy

##### 3.4.4.1 Mixed interventions

Lin et al. (2024) evaluated a diversified health-promoting model, which combined physical exercise and psychological support, and a conventional health-promoting model, which included postoperative standard care. Self-efficacy was assessed using the Self-Efficacy Rehabilitation Outcome (SER) scale, which measures coping self-efficacy and physical exercise self-efficacy. In the diversified health-promoting group, SER scores significantly improved from baseline to postoperative day 3, and further to discharge. While both groups began at comparable baselines (p = 0.98), the diversified health-promoting group demonstrated statistically significant improvements in self-efficacy by day 3 (p = 0.04) and prior to discharge (p < 0.001).

### 3.5 Cervical spine impairments

A complete summary of cervical spine impairments is presented in Tables S2 and S4.

#### 3.5.1 Neurological function

##### 3.5.1.1 Active interventions

Rahman et al. (2024) evaluated postoperative neurological function using the modified Japanese Orthopaedic Association (mJOA) score following a postsurgical rehabilitation program that included occupational therapy and physical therapy. Both interventions were associated with improvement from preoperative to 12-month postoperative scores. Specifically, OT was significantly associated with greater improvement in mJOA scores (p = 0.019), whereas PT did not show a statistically significant effect (p = 0.60).

##### 3.5.1.2 Passive interventions

Cheung et al. (2019) evaluated neurological recovery using mJOA score in patients who either wore a postoperative cervical collar or did not. Both groups showed improvement in mJOA scores from preoperative to 12-month postoperative timepoints, though there was no significant difference between the collar and no-collar groups (p = 0.636). Iizuka et al. (2005) evaluated neurologic recovery using JOA scores in patients who wore a cervical collar for 8 weeks versus 4 weeks postoperatively. Both groups showed improvement, but there was no significant difference in recovery between the two durations of collar use (p > 0.47).

##### 3.5.1.3 Mixed interventions

Farrokhi et al. (2024) assessed neurological function using the mJOA scale following an intervention of physiotherapy combined with repetitive transcranial magnetic stimulation (rTMS) versus physiotherapy alone. Both groups showed statistically significant improvements post-intervention, with greater improvement observed in the physiotherapy + rTMS group (p = 0.000) compared to the physiotherapy-alone group (p = 0.001). Lin et al. (2024) assessed neurological recovery using the JOA score in patients who received a diversified health-promoting model versus those who received conventional health promotion. While no significant difference in JOA scores was observed between groups at baseline (p = 0.703) or 3 days post-intervention (p = 0.975), a significant improvement favoring the diversified health-promoting model was observed prior to discharge (p = 0.001). Yue and Liu (2021) also evaluated JOA scores, comparing patients receiving timeliness incentive nursing to those who received routine nursing care. Although both groups improved significantly from baseline (p < 0.001), the intervention group demonstrated significantly greater improvement in JOA scores compared to the control group (p < 0.001). Tamai et al. (2023) evaluated neurological recovery using the JOA scores in patients who followed a multidisciplinary postoperative rehabilitation protocol compared to those who received standard care. Both groups showed improvement in JOA scores at the 1-year follow-up, with a statistically significant improvement observed only in the multidisciplinary protocol group (p = 0.040).

#### 3.5.2 Cervical muscle strength

##### 3.5.2.1 Active interventions

Uehara et al. (2022) examined neck muscle strength following cervical surgery and implemented early rehabilitation, initiating isometric neck extension and cervical range of motion exercises starting on postoperative day two. Both the exercise (isometric strength and range of motion) and control (no exercise) groups experienced a decline in neck extension strength at two weeks postoperatively. However, by three months, only the exercise group showed a statistically significant recovery above baseline, with significant time effects (p < 0.001). Overall improvements in extension strength were significantly associated with reduced axial pain at three months (p = 0.004). Neck flexion strength also significantly improved in both groups from baseline to three months.

#### 3.5.3 Cervical range of motion

##### 3.5.3.1 Passive interventions

Cheung et al. (2019) evaluated cervical range of motion (ROM) in six directions (flexion, extension, lateral flexion, and rotation) in two groups: cervical collar vs. no-collar. Both groups experienced early postoperative declines in ROM and recovery by 12 months. Across all directions of motion, both groups showed similar trends of improvement or minor decline, with no statistically significant differences between groups at 12 months (all p-values >0.193). Iizuka et al. (2005) evaluated cervical ROM in two groups: one wearing a cervical collar for 8 weeks and another for 4 weeks. While cervical alignment was similarly preserved in both groups, differences emerged in ROM. The 4-week collar group maintained significantly greater total cervical ROM compared to the 8-week group (p < 0.03), particularly in flexion and extension.

##### 3.5.3.2 Mixed interventions

Yue and Liu (2021) assessed cervical ROM following a timeliness incentive nursing intervention compared to routine nursing care. Before the intervention, there were no significant differences in ROM between the two groups (p > 0.05). After the intervention, both groups showed significant improvements (p < 0.001), with the intervention group exhibiting significantly greater gains than the control group (p < 0.001).

#### 3.5.4 Cervical spine alignment

##### 3.5.4.1 Active interventions

Uehara et al. (2022) investigated the impact of early cervical exercise following laminoplasty on cervical alignment, focusing on C2-C7 lordosis, C7 slope, and sagittal vertical axis (SVA). Both the exercise (i.e., isometric strength and range of motion) and control (i.e., no exercise) groups demonstrated reductions in C2-C7 lordosis and C7 slope over the three-month period. While there were significant time effects for lordosis (p < 0.001) and slope (p = 0.023), there were no significant group effects or group × time interactions for either outcome. Both groups showed a minor increase (∼1 mm) in C2-C7 SVA, with no significant changes, although the group effect approached significance (p = 0.06).

#### 3.5.5 Motor function

##### 3.5.5.1 Mixed interventions

Catz et al. (2024) implemented a comprehensive inpatient rehabilitation program following surgery for DCM. This multidisciplinary intervention included physical therapy, occupational therapy, and standard medical care. Patients demonstrated statistically significant improvements in motor function, with ASIA Motor Scores increasing from admission to discharge (p < 0.001). Additionally, Farrokhi et al. (2024) compared outcomes between patients receiving physiotherapy alone and those receiving physiotherapy combined with repetitive transcranial magnetic stimulation (rTMS). Both groups showed statistically significant improvements in lower and total motor scores postoperatively, but improvements were greater in the combined group (all p ≤ 0.004).

#### 3.5.6 Muscle tone

##### 3.5.6.1 Mixed interventions

Farrokhi et al. (2024) assessed lower limb muscle tone using the Modified Ashworth Scale in patients who received physiotherapy combined with repetitive transcranial magnetic stimulation (rTMS) versus physiotherapy alone. Both groups showed improvement, with a significantly greater reduction in muscle tone observed in the physiotherapy + rTMS group (p = 0.003).

### 3.6 Functional outcomes

A complete summary of functional outcomes is presented in Tables S2 and S4.

#### 3.6.1 Active interventions

Cheng et al. (2020) evaluated mobility using the Timed Up and Go (TUG) test in postoperative DCM patients who received 4 weeks of perturbation-based balance training (PBT) versus healthy controls with no intervention. The DCM group was significantly slower pre-training (p < 0.01) but improved significantly after training (p < 0.01), with no significant between-group difference post-intervention (p = 0.15).

#### 3.6.2 Mixed interventions

Lin et al. (2024) evaluated the grip strength of the affected limb following a diversified health-promoting model compared to conventional health promotion (control group). No significant differences were observed between groups at baseline or 3 days post-intervention (p = 0.998), but by discharge, the intervention group showed significantly greater grip strength than the control group (p = 0.041). Functional independence was also assessed using the Barthel Index. No significant differences were found between groups at baseline (p = 0.661) or 3 days post-intervention (p = 0.753), but by discharge, the intervention group showed significantly greater improvements than the control group (p = 0.003). Farrokhi et al. (2024) assessed functional mobility using the Nurick Scale in two groups: one receiving physiotherapy combined with rTMS, and the other receiving physiotherapy alone. Both groups improved post-intervention, but the combined intervention group showed a greater reduction in Nurick scores (p = 0.001).

### 3.7 Postoperative complications

A complete summary of postoperative complications is presented in Tables S2 and S4.

#### 3.7.1 Passive interventions

Cheung et al. (2019) evaluated cervical collar vs. no cervical collar following DCM decompressive surgery. No complications were reported with any of their participants.

#### 3.7.2 Active interventions

Rahman et al. (2024) evaluated postoperative complications following a postsurgical rehabilitation program that included occupational therapy and physical therapy. Postoperative complications included one case of ileus managed conservatively, one pulmonary embolism requiring readmission, and one hardware revision for C7 pedicle screw repositioning across both groups.

#### 3.7.3 Mixed interventions

Yue and Liu (2021) evaluated the impact of a timeliness incentive nursing model, which combined elements of active mobilization support, structured care timelines, and patient motivation strategies. The timeliness incentive nursing group experienced significantly earlier out-of-bed activity and shorter hospital stays compared to controls (p < 0.001 for both). Additionally, the timeliness incentive nursing group had a significantly lower complication rate (3.85%) versus the control group (17.31%, p = 0.026) (Table S4). Reductions were observed across multiple complications, including wound edema, deep vein thrombosis, and spastic paralysis.

### 3.8 Evidence summary

The quality of evidence was very low to low, preventing a definitive conclusion from being drawn regarding the role of rehabilitation after DCM surgery. With respect to patient-reported outcomes, functional outcomes, and postoperative complications, the body of evidence was limited by serious concerns related to risk of bias, imprecision, and potential publication bias, yielding very low quality of evidence (Table 3). Concerning cervical spine impairment outcomes, there were serious limitations regarding risk of bias and publication bias, yielding low-quality evidence (Table 3).

## 4 Discussion

### 4.1 Comparison with other systematic reviews

The present review focuses exclusively on postoperative rehabilitation in DCM, offering a targeted analysis of how intervention type and structure impact recovery. Our review identified 10 studies with 766 participants undergoing postoperative rehabilitation for DCM. Given the low quality of evidence among the studies, a definitive conclusion on the role of postoperative rehabilitation was difficult to ascertain. However, key rehabilitation targets, which showed emerging evidence were identified, including neurological function, quality of life, and self-efficacy. Prior reviews in this field have either focused on broader populations or lacked sufficient data on rehabilitation following surgery. Badran et al. found only one low-quality retrospective study addressing postoperative physiotherapy for DCM, concluding that there was insufficient evidence to make any recommendations (Badran et al., 2018). Lantz et al. evaluated postoperative physical therapy in cervical spine disorders more broadly, but did not isolate DCM as a distinct population, and ultimately found the quality of evidence too low to draw conclusions due to lack of control groups and inconsistent protocols (Lantz et al., 2021). Ling et al. expanded the scope to include patients with cervical spondylosis undergoing fusion, again not focusing on DCM specifically, and reported mixed findings with no consistent benefit of rehabilitation strategies (Ling et al., 2023). A recent systematic and scoping review of United Kingdom-based patient information found that, despite growing recognition of DCM's life-altering impact, evidence-based guidelines for pre-and postoperative rehabilitation remain lacking (Smith et al., 2025).

### 4.2 Patient-reported outcome measures

#### 4.2.1 Effect of postoperative rehabilitation on pain

Pain outcomes following surgery for DCM improve across rehabilitation types, but the magnitude and consistency of relief vary. Active rehabilitation generally supports the gradual reduction of postoperative pain, although its effectiveness depends on the type of intervention. Pain commonly decreases naturally in the early postoperative period, regardless of specific treatments, reflecting the body's intrinsic healing processes. While some active therapies, such as targeted exercises, may contribute to improved pain outcomes, interventions focused solely on balance or other isolated modalities appear insufficient to address the complex, multifactorial nature of postoperative pain in DCM (Rahman et al., 2024; Farrokhi et al., 2024; Cheung et al., 2019). Passive interventions, specifically cervical collar use, provided short-term relief by immobilizing the neck and reducing early axial pain following surgery (Cheung et al., 2019). However, their benefits seem to diminish over time, showing no lasting difference compared to non-use. While they may provide temporary mechanical support and limit movement, they do not address underlying neuromuscular and inflammatory factors that contribute to persistent postoperative discomfort. Mixed interventions demonstrated the most robust improvements in pain outcomes, particularly when combining physical rehabilitation with patient-centered care strategies, though not all interventions were statistically significant (Yue and Liu, 2021; Tamai et al., 2023). The effectiveness of mixed interventions likely stems from targeting pain from multiple angles, including neuromuscular, behavioral, and emotional, creating a more comprehensive pain management effect. Raising awareness about the complexity of DCM and its post-surgical sequelae, including chronic pain, is crucial for optimizing rehabilitation approaches and outcomes (Wardropper et al., 2025).

#### 4.2.2 Effect of postoperative rehabilitation on quality of life

Mixed interventions, which integrated both active (e.g., exercise) and passive (e.g., education, medical support) elements, consistently demonstrated the strongest improvements in health-related quality of life following cervical spine surgery. Multimodal programs (i.e., occupational therapy, psychosocial support) demonstrated a significant improvement in the physical and social aspects of quality of life (Rahman et al., 2024; Yue and Liu, 2021). Similarly, the positive trends observed in Tamai et al. support the potential for targeted interventions to address self-care abilities (Tamai et al., 2023). The selective improvement in self-care may reflect the specific and immediate functional benefits of physical rehabilitation strategies targeting daily tasks, such as dressing, bathing, and feeding. Individuals with DCM experience some of the lowest quality of life scores among chronic disease populations, as demonstrated by SF-36 scores. By simultaneously addressing pain, physical impairments and psychosocial challenges, postoperative rehabilitation may play a central role in improving quality of life, thus enabling participation in daily and social activities.

#### 4.2.3 Effect of postoperative rehabilitation on disability

Postoperative rehabilitation interventions demonstrated a general trend toward reduced disability in patients with DCM, although statistical significance was often lacking. Active rehabilitation interventions appear to support improvements in disability following DCM surgery; however, the evidence remains inconclusive. While some programs incorporating physical and occupational therapy or balance training reported positive trends in functional outcomes, these often did not reach statistical significance (Rahman et al., 2024; Uehara et al., 2022; Cheng et al., 2020). Although patients typically experience some improvement after surgery, full recovery is rare because the spinal cord has a limited capacity to heal, and residual disability often reflects irreversible damage (Badhiwala and Wilson, 2018; Davies et al., 2022). As emphasized by AO Spine RECODE-DCM research priorities, optimizing surgical treatment timing through expediting diagnosis and coordinated management is necessary to improve outcomes, including disability (Davies et al., 2022).

#### 4.2.4 Effect of postoperative rehabilitation on self-efficacy

Early postoperative rehabilitation that integrates exercise with psychological and behavioral support can improve patient self-efficacy (Lin et al., 2024). Improvements in self-efficacy observed within days of surgery suggest that early intervention may set a positive trajectory for recovery, reinforcing both engagement and motivation. Given the well-established link between self-efficacy, engagement and recovery success, incorporating strategies to build confidence should be considered a routine component of postoperative care planning (Gangwani et al., 2022). Supporting patients early in their rehabilitation journey may foster a compounding effect, amplifying recovery over time. However, successful implementation of early rehabilitation strategies may also depend on the quality of preoperative education. A recent systematic and scoping review of United Kingdom National Health Service patient information on DCM surgery found that while patients are routinely informed about surgical risks and return-to-activity timelines, there is limited guidance on structured postoperative exercise (Smith et al., 2025), which may affect patient readiness and engagement in rehabilitation.

### 4.3 Cervical spine impairments

#### 4.3.1 Effect of postoperative rehabilitation on neurological function

Neurological function improved across all intervention types, but the degree and consistency of improvement varied depending on the type of rehabilitation protocol. Active interventions led to measurable improvements in neurological outcomes, particularly when they targeted specific functional domains. Interventions that engaged patients in task-specific movement, such as occupational therapy, were particularly effective, likely due to their focus on fine motor skills and activities of daily living, domains commonly affected by DCM (Rahman et al., 2024). In contrast, passive interventions (i.e., cervical collars) may help manage early postoperative pain or prevent movement-related complications, their contribution to long-term neurological improvement appears to be minimal (Cheung et al., 2019; Iizuka et al., 2005). Despite providing early mechanical protection, collars do not stimulate the neuromuscular system or promote relearning of movement patterns, mechanisms critical to neurological restoration. Mixed rehabilitation strategies combining physical therapy with neuromodulatory, physical, psychological or motivational components demonstrated the most consistent improvements across neurological outcomes. Programs that integrated repetitive transcranial magnetic stimulation (rTMS), structured multidisciplinary care, or behaviorally-informed nursing strategies outperformed unimodal approaches (Lin et al., 2024; Farrokhi et al., 2024; Yue and Liu, 2021; Tamai et al., 2023). Multimodal interventions likely achieve superior results by addressing multiple recovery pathways simultaneously, motor, cognitive, and psychosocial, highlighting the value of a comprehensive rehabilitation model in addressing DCM's complex functional impairments (Tetreault et al., 2022).

#### 4.3.2 Effect of postoperative rehabilitation on cervical muscle strength

Muscle strength recovery following surgery appears to benefit from early mobilization and active rehabilitation. Initiating neck-specific exercises, including isometric extension and range of motion, as early as postoperative day two can lead to significant improvements in neck extensor strength (Uehara et al., 2022). Strength improvements (i.e., extension) were significantly associated with reductions in axial pain, suggesting both functional and symptomatic benefits of early muscular reactivation. While improvements in neck flexion were comparable between individuals who completed exercise versus those who did not, the greater improvements in extension likely reflect the specificity of the exercise protocol. Early, targeted strengthening plays a key role in improving neuromuscular recovery after surgery. However, a lack of long-term follow-up and mechanistic muscle data (e.g., ultrasound, EMG) limits understanding of underlying structural changes and functional carryover.

#### 4.3.3 Effect of postoperative rehabilitation on cervical range of motion

Postoperative recovery of cervical range of motion (ROM) appears to follow a natural trajectory, but specific rehabilitation strategies can influence the speed and extent of improvement (Cloney et al., 2018; Cheung et al., 2019; Yue and Liu, 2021; Iizuka et al., 2005). Interventions that combine physical rehabilitation with behavioral or motivational support may further improve recovery, accelerating improvements beyond the natural course of healing (Yue and Liu, 2021; Nicolau et al., 2022). Additionally, shorter durations of cervical collar use (e.g., 4 weeks vs. 8 weeks) are associated with better preservation of extension and overall cervical ROM (Iizuka et al., 2005; Wang et al., 2022). Prolonged immobilization may hinder recovery by contributing to joint stiffness and muscle disuse, highlighting the need to balance protective measures with timely collar weaning, early mobilization and structured rehabilitation in postoperative care (Cheung et al., 2019; Ackland and Cameron).

#### 4.3.4 Effect of postoperative rehabilitation on cervical spine alignment

While early active rehabilitation supports neuromuscular recovery after surgery, its short-term impact on cervical spinal alignment appears limited. Short-duration exercise programs may not be sufficient to alter sagittal alignment parameters such as cervical lordosis, C7 slope, or C2–C7 sagittal vertical axis (Uehara et al., 2022). Observed temporal changes for lordosis and C7 slope likely reflect natural postoperative adaptation rather than intervention-specific effects. Notably, C2-C7 SVA values showed minimal change following cervical exercise but trended towards statistical significance, suggesting a potential benefit in preserving spinal alignment. Nevertheless, spinal alignment changes appear to be influenced more by surgical technique and structural factors rather than postoperative rehabilitation alone (Barbosa et al., 2023; Metkar et al., 2024).

#### 4.3.5 Effect of postoperative rehabilitation on motor function

Motor function improvements appear greater when rehabilitation integrates physical therapy with neurological and behavioral strategies. Both Catz et al. and Farrokhi et al. highlight the benefits of mixed postoperative rehabilitation strategies in improving motor function following decompressive surgery for DCM (Farrokhi et al., 2024; Catz et al., 2024). Combining repetitive transcranial magnetic stimulation (rTMS) with physiotherapy led to significantly greater improvements in lower and total motor scores compared to physiotherapy alone (all p ≤ 0.004), suggesting that neuromodulation may improve neuroplasticity and motor recovery beyond what traditional rehabilitation achieves (Farrokhi et al., 2024). Motor function recovery may be optimized through interventions that integrate physical, neurological, and behavioral components, rather than relying solely on conventional physical therapy.

#### 4.3.6 Effect of postoperative rehabilitation on muscle tone

Combining neuromodulation with physiotherapy may help manage lower limb spasticity after cervical spine surgery. Farrokhi et al. found that adding repetitive transcranial magnetic stimulation (rTMS) to physiotherapy reduced lower limb spasticity more than physiotherapy alone, suggesting a role for targeted tone modulation to improve functional recovery (Farrokhi et al., 2024; Wolpaw and Thompson, 2023). Although the nearly one-point reduction in MAS scores did not reach the threshold for clinical significance, it reflects a change that may still contribute to greater ease of movement and reduced discomfort in daily activities. The incorporation of tone-reducing strategies into rehabilitation protocols, particularly for patients presenting with heightened lower limb spasticity after cervical decompression surgery, may be beneficial (Ghai et al., 2013).

### 4.4 Effect of postoperative rehabilitation on function

Structured postoperative rehabilitation appears to support functional recovery, particularly when active and mixed interventions are applied early. Perturbation-based balance training significantly improved mobility among patients with DCM, as evidenced by faster Timed Up and Go (TUG) times post-training (p < 0.01) and a normalization of performance relative to healthy controls (p = 0.15) (Cheng et al., 2020). Dynamic balance challenges may help restore ambulatory function and reduce postoperative gait deficits (Chang et al., 2024). An early diversified health-promoting model improved Barthel Index scores (p = 0.003) and grip strength (p = 0.041) by discharge, suggesting that early rehabilitation not only restores independence in activities of daily living but may also enhance neuromuscular recovery in the upper limbs (Lin et al., 2024). These improvements, observed within a short postoperative window, reflect the sensitivity of functional measures to early intervention (Li et al., 2024). Similarly, the incorporation of repetitive transcranial magnetic stimulation (rTMS) to physiotherapy reduced Nurick Grade scores, suggesting that neuromodulation may augment the impact of traditional functional training (Evancho et al., 2023). Functional outcomes are responsive to timely, multimodal rehabilitation approaches, particularly when both neurophysiological and task-specific strategies are used early in recovery. Further research is needed to better understand how DCM impairs function, contributes to disability, and negatively affects quality of life (Tetreault et al., 2022).

### 4.5 Effect of postoperative rehabilitation on complications

Although our review focused on rehabilitation, the potential impact of postoperative complications on rehabilitation uptake and outcomes should not be overlooked. In the context of adult spinal deformity, a recent systematic review found that increased surgeon experience significantly reduced complication rates, highlighting the importance of surgical factors in postoperative recovery (Anastasio et al., 2024). While the evidence is specific to spinal deformity surgery, it suggests that complication burden, potentially influenced by surgical expertise, may indirectly affect the timing and effectiveness of postoperative rehabilitation.

Integrated, protocol-driven interventions may reduce both clinical and economic burdens by preventing common postoperative complications (Yue and Liu, 2021). The integration of early active mobilization, structured care timelines, and patient motivation strategies achieved significantly earlier ambulation, shorter hospital stays, and lower complication rates, including wound edema, hematoma, and deep vein thrombosis. Such improvements in mobilization and complication rates align with the enhanced recovery after surgery (ERAS) principles (Naftalovich et al., 2022). Similar patterns are evident in the orthopedic and spine surgery literature, where early rehabilitation has been consistently linked to improved outcomes and recovery trajectories (Elsarrag et al., 2019; Burgess and Wainwright, 2019; Lantz et al., 2023). Reviews support the role of early mobilization in promoting functional recovery without increasing complication rates (Elsarrag et al., 2019; Burgess and Wainwright, 2019). Similarly, a retrospective study found no significant changes in complications when postoperative physical therapy for cervical spine surgery was initiated at 2, 8, or 12 weeks, suggesting that earlier rehabilitation may be safely implemented (Lantz et al., 2023). As healthcare systems shift toward value-based care, such models may offer scalable strategies to optimize recovery while reducing hospital resource use.

### 4.6 Limitations

This systematic review synthesizes current evidence on postoperative rehabilitation for DCM, but several limitations should be acknowledged when interpreting the findings. First, a lack of standardized, detailed rehabilitation protocols across many included studies limits the reproducibility of interventions and complicates cross-study comparisons. While most studies identified the type of exercises or general therapeutic approach used, they often failed to specify the frequency, intensity, progression, or session duration in sufficient detail to allow for replication. Second, differences in the timing of rehabilitation initiation, type of decompression, and extent of spinal cord involvement may have affected recovery trajectories, making it more difficult to isolate the impact of rehabilitation itself. Lastly, the majority of included studies had a high risk of bias, largely due to unclear randomization, lack of assessor blinding, and absence of published protocols.

### 4.7 Future research

Future research should establish and report standardized rehabilitation protocols with clear details on exercise type, frequency, intensity, duration, and progression to improve consistency and comparability across studies. Studies should also incorporate validated DCM-specific outcome measures (e.g., mJOA, NDI, cervical range of motion, neck and grip strength, gait speed, etc.) to improve clinical relevance and accurately assess rehabilitation effects. The AO Spine RECODE-DCM initiative identified several research priorities directly supporting this need, including defining the role of rehabilitation and identifying the most meaningful outcome measures (Chauhan et al., 2025; Davies et al., 2022). Prioritizing these gaps is essential to improving care quality and enhancing recovery for individuals living with DCM.

## 5 Conclusion

Overall, this systematic review highlights that postoperative rehabilitation may offer meaningful benefits for individuals recovering from DCM decompressive surgery, though the evidence remains weak. Despite these promising trends, heterogeneity in protocols, outcome measures, and study quality limits definitive conclusions. Standardized, well-described interventions and consistent outcome reporting will be essential for advancing the evidence base and optimizing rehabilitation strategies for individuals with DCM.

## Author contributions

Conceptualization, C.M., J.M.L., D.B.A., M.F.; Data curation, C.M., A.K.; Formal analysis, C.M., A.K.; Investigation, C.M., A.K.; Methodology, C.M., J.M.L., D.B.A., M.F.; Project administration, C.M., M.F.; Supervision, M.F.; Writing—original draft, C.M., A.K., M.F.; Writing—review and editing, C.M., A.K., J.M.L., R.V.C., D.B.A., M.F. All authors have read and agreed to the published version of the manuscript.

## Ethical approval statement

This systematic review used only previously published data and did not require ethics approval.

## Funding statement

This research did not receive any specific grant from funding agencies in the public, commercial, or not-for-profit sectors.

## Declaration of competing interest

The authors declare that they have no known competing financial interests or personal relationships that could have appeared to influence the work reported in this paper.

## Figures and Tables

**Table 1:** Inclusion and Exclusion Criteria (PICOS Framework)

PICOS Element	Inclusion Criteria	Exclusion Criteria
Population	Adults diagnosed with DCM confirmed by imaging (e.g., MRI) or clinical criteria (e.g., mJOA) following surgical intervention.	Populations without confirmed DCM diagnosis.
Intervention	Postoperative rehabilitation including but not limited to physical therapy, exercise, flexibility training, or mixed rehabilitation in hospital, home, or community settings.	Studies lacking detailed rehabilitation protocols or unclear methodologies.
Comparison	No rehabilitation, usual care, pharmacological treatment, or alternative interventions such as rTMS.	N/A.
Outcomes	Patient-Reported Outcome Measures: pain. quality of life, disability, self-efficacy. Cervical spine impairments: neurological function, cervical muscle strength, cervical range of motion, cervical spine alignment, motor function, muscle tone. Functional outcomes: grip strength, Timed Up and Go, Nurick Grade Scale. Postoperative complications.	Studies not reporting relevant outcomes.
Study Design	Randomized controlled trials (RCT), quasi-RCTs, longitudinal studies, cohort studies, non-randomized studies; ≥8 participants/group; peer-reviewed; English or French.	Animal, cadaveric, or basic science studies, case reports, reviews, letters, editorials, surveys, diagnostic/prognostic studies, opinion pieces, non-peer-reviewed articles.

**Table 2:** Summary of Primary Outcomes from the included Articles

Study	Primary Outcome	Duration + Follow-Up Time	Intervention Group	Control Group	P-value
Rahman et al. (2024)	Function	Duration: Not specified FU: 12 months	PT/OT mJOA: 14.2 ± 2.4 NDI: 24.9 ± 18.5 SF-36: 42.3 ± 11.0	No control group.	N/A
Catz et al. (2024)	Motor function	Duration: 72 ± 40 days FU: N/A	Comprehensive rehab AMS: 85.0 ± 15.3	No control group.	N/A
Uehara et al., 2022	Pain	Duration: 3 months FU: 3 months	Cervical exercise VAS: 25.3 ± 23.1	Control (no exercise) VAS: 20.6 ± 18.3	p = 0.61
Tamai et al., 2023	QoL	Duration: 6 months FU: 1 year	MA EQ-5D-5L: 0.79 ± 0.17	SC EQ-5D-5L: 0.72 ± 0.21	p = 0.15
Lin et al. (2024)	Self-efficacy	Duration: ∼8 days FU: N/A	DHPM SER: 81.82 ± 6.95	CHPM SER: 75.53 ± 7.66	p < 0.001
Farrokhi et al. (2024)	Neurological function	Duration: 2 weeks FU: N/A	PT + rTMS UEMS: 44.31 ± 4.85 LEMS: 45.23 ± 5.00 mJOA: 14.38 ± 2.46 Ashworth: 0.85 ± 0.55 Nurick: 1.54 ± 0.09	PT UEMS: 43.92 ± 7.53 LEMS: 45.08 ± 7.05 mJOA: 15 ± 2.5 Ashworth: 1.23 ± 0.43 Nurick:2.07 ± 0.64	UEMSRR p = 0.008 LEMSRR p = 0.15 mJOA p = 0.002 Ashworth p = 0.02 Nurick p = 0.006
Cheung et al. (2019)	Pain	Duration: 3 weeks FU: 12 months	CC VAS: 1.1 ± 1.7	Control VAS: 1.7 ± 2.4	p = 0.61
Cheng et al. (2020)	Balance	Duration: 4 weeks FU: N/A	PBT, no numerical values provided.	Healthy controls, no intervention provided.	COP p = 0.34 COM forward p = 0.11 COM backward p = 0.34
Yue and Liu (2021)	Pain, cervical spine function, cervical ROM, OOB activity	Duration: 2 months FU: N/A	TIN, no numerical values provided.	Control (RN), no numerical values provided.	p < 0.05
Iizuka et al. (2005)	Total flexion + extension ROM	Duration: 4–8 weeks FU: N/A	CC 8W: 24.2° ± 12.9° CC 4W: 35.3° ± 12.7°	No control group.	p < 0.03

FU, follow-up; mJOA, modified Japanese Orthopaedic Association; NDI, Neck Disability Index; AMS, American Motor Score; VAS, Visual Analog Scale; MA, multidisciplinary approach; SC, standard care; QoL, quality of life; EQ-5D-5L, EuroQoL 5-dimension 5-level; DHPM, diversified health promoting model; CHPM, conventional health promoting model; SER, Self-efficacy rehabilitation outcome; PT + rTMS, physiotherapy plus repetitive transcranial magnetic stimulation; PT, physiotherapy; UEMS, upper extremity motor score; LEMS, lower extremity motor score; UEMSRR, upper extremity motor score recovery rate; LEMSRR, lower extremity motor score recovery rate; CC, cervical collar; PBT, pertubation-based balance training; COP, Center of pressure; COM, Center of mass; OOB, TIN, timeliness incentive nursing; RN, routine nursing; out-out-bed; JOA, Japanese Orthopaedic Association; ROM, range of motion.

**Table 3:** Summary of Quality of Evidence

Finding	Risk of Bias	Indirectness	Inconsistency	Imprecision	Publication Bias	Quality of Evidence
Patient-reported outcomes	Serious limitations, downgraded 2 level	No serious limitations	No serious limitations	Serious limitation, downgraded 1 level	Serious limitation, downgraded 1 level	Very low
Cervical Spine Impairment	Serious limitations, downgraded 2 level	No serious limitations	No serious limitations	No serious limitations	Serious limitation, downgraded 1 level	Low
Functional Outcomes	Serious limitations, downgraded 2 level	No serious limitations	No serious limitations	Serious limitation, downgraded 1 level	Serious limitation, downgraded 1 level	Very low
Postoperative Complications	Serious limitations, downgraded 1 level	No serious limitations	No serious limitations	Serious limitation, downgraded 1 level	Serious limitation, downgraded 1 level	Very low

## Data Availability

All extracted data are available upon request.
