# Supplementary material for: The effect of postoperative rehabilitation on outcomes in patients with degenerative cervical myelopathy (DCM): A systematic review
Source: Brain Spine. 2026 Feb 2;6:105956. doi: 10.1016/j.bas.2026.105956 (PMC12906147; doi:10.1016/j.bas.2026.105956)
Supplement: Multimedia component 1 [file mmc1.docx]

**Table S1.** Literature Search Strategy.

| **Category** | **Search Term** |
| --- | --- |
| Degenerative cervical myelopathy (DCM) | "Cervical myelopathy" OR DCM OR "Cervical spinal stenosis" OR "Degenerative spine disease" OR "Cervical spondylosis" OR "Spinal cord compression" OR "cervical spinal cord compression" OR "Cervical degenerative disease" OR "Cervical spine disorder" OR "Degenerative cervical myelopathy" OR "Cervical compression" OR "cervical disc disease" OR "cervical disk disease" OR "cervical discopathy" OR "cervical spine disease" OR "degenerative cervical spondylosis" OR "cervical spine disorder" OR "spondylotic myelopathy" OR "cervical compression syndrome" OR "cervical spinal stenosis" |
|  | And |
| Surgery / Post-Operative | (Anterior cervical discectomy" OR "Anterior cervical discectomy and fusion" OR "cervical disc removal" OR "Anterior cervical corpectomy" OR "Anterior cervical corpectomy and fusion" OR "Anterior cervical disc replacement" OR "Posterior cervical laminectomy" OR "Posterior cervical laminoplasty" OR "Cervical disc replacement" OR "Combined anterior-posterior surgery" OR "cervical fusion" OR "cervical decompression" OR postsurgery OR post-surgery OR postsurgical OR postoperative OR post-operative OR perioperative OR peri-operative) OR ("Spinal Cord Diseases/surgery"[Mesh] OR "Spinal Cord Compression/surgery"[Mesh] OR "Cervical Vertebrae/surgery"[Mesh]) |
|  | And |
| Post-Intervention Rehabilitation | (exercise OR fitness OR strength OR "physical activity") AND (therapy OR training OR rehabilitation OR intervention OR treatment) OR (physiotherapy OR physiotherapeutic OR rehabilitative OR rehabilitation OR "physical therapy" OR "rehab") OR ("Spinal Cord Diseases/rehabilitation"[Mesh] OR "Spinal Cord Compression/rehabilitation"[Mesh]) |

**Table S2**. Demographics of Included Studies.

| **Reference** | **Study Design** | **Surgery** | **Postoperative Rehabilitation** | **Comparison** | **Demographics** | **Time points** | **Outcomes** |
| --- | --- | --- | --- | --- | --- | --- | --- |
| Rahman et al. 2024 | Prospective cohort study | Elective first-time spine surgery for DCM | Postsurgical rehabilitation including OT & PT | PT vs. OT  Early rehabilitation (<42 days) vs. delayed rehabilitation (>42 days | Sample size: 66 patients; Age (mean, SD): 55 (11) years; Sex: 37 (56%) female; Smoking status: 60 (91%) nonsmokers; Diabetes status: 59 (89%) nondiabetic | Pre-operative  12-month post-operative | mJOA  NDI  SF-36 PCS |
| Catz et al. 2024 | Retrospective cohort study | Spinal surgery for degenerative cervical spine | Comprehensive inpatient rehabilitation program | None. | Sample size: 116 patients  Age (mean, SD): 60.6 (12) years ; Male (%): 73.3% | Admission (t=0)  Discharge (t=72 ± 40 days) | AMS  SCIM-III  dSCIM-III  SCI-ARMI |
| Uehara et al. 2022 | Randomized controlled trial | Cervical laminoplasty | Neck extension isometric muscle strengthening and cervical ROM exercises from day 2 post-op for 3 months | Cervical exercise group vs. control group (no exercise) | Sample size: 61 patients (Exercise group: 33, Control: 28); Age (mean, SD): 71.6 (10.5) years; Male (%): ~71% | Baseline  2-week follow-up  3-month follow-up | VAS  JOACMEQ  Neck muscle strength  Cervical spine alignment (C2-7 lordosis angle, C7 slope, C2-7 sagittal vertical axis)  Cervical ROM |
| Tamai et al. 2022 | Prospective cohort study | Cervical laminoplasty | MA | SC vs. MA group | Sample size: 171 patients (SC: 140, MA: 31); Age (mean, SD): SC: 73.5 (7.0) years, MA: 72.2 (6.9) years | Pre-operative  1-year post-operative | JOA  VAS  EQ-5D-5L  NDI  JOACMEQ |
| Lin et al. 2024 | Randomized controlled trial | Anterior cervical discectomy and fusion | DHPM  CHPM | DHPM vs.  CHPM | Sample size: 107 patients (DHPM: 55, CHPM: 52); Age (mean, SD): DHPM: 58.87 (9.41) years, CHPM: 58.25 (8.60) years | Pre-intervention  Day 3 post-operative  Prior to discharge | SER  Grip strength  JOA  Barthel index |
| Farrokhi et al. 2024 | Randomized controlled trial | Cervical laminectomy | PT alone vs.  PT + rTMS | PT vs.  PT + rTMS | Sample size: 52 patients (26 per group); Age (mean, SD): PT + rTMS: 60.54 (6.55) years, PT: 54.08 (14.78) years; Male (%): 80% | Before and after intervention (2-weeks) | UEMS  LEMS  mJOA  Ashworth scale  Nurick scale |
| Cheung et al. 2019 | Randomized controlled trial | Single-door laminoplasty | Cervical collar vs. no collar | Collar vs. No-collar | Sample size: 35 patients (Collar: 16, No Collar: 19); Age (mean, SD): 64.9 (11.4) years | Preoperatively postoperative 1, 2, 3, and 6 wk, and 3, 6, and 12 months | Cervical ROM  VAS  SF-36  NDI  mJOA |
| Cheng et al. 2020 | Prospective non-randomized clinical trial | Cervical decompression surgery | PBT | DCM group vs. healthy controls | Sample size: 15 DCM patients, 14 healthy controls; Age (mean, SD): DCM: 64.0 (5.3) years, Controls: 67.4 (5.9) years; Male (%): 67% | Before and after intervention (4-weeks) | Center of pressure  Center of mass  Variance and reaction time to balance perturbation  Gait speed  Timed up and go  VAS  NDI  JOACMEQ-LEF |
| Yue & Liu 2021 | Randomized controlled trial | Various cervical spine surgeries | TIN model vs.  RN | TIN model vs.  RN | Sample size: 104 patients (TIN: 52, Control: 52); Age (mean, SD): Control: 58.3 (6.1) years, TIN: 60.7 (7.3) years | Baseline  2 months post-intervention | Out-of-bed activity time  Length of hospital stay  VAS  JOA  Cervical ROM  SF-36  Complications  Nursing satisfaction |
| Iizuka et al. 2005 | Retrospective cohort study | Cervical laminoplasty | Cervical collar | Cervical collar for 4 weeks vs. 8 weeks | Sample size: 51 patients (4W: 26, 8W: 25); Age (mean, SD): 4W: 60.6 years (range 37-78), 8W: 61.4 years (range 36-79) | 4 weeks and 8 weeks post-operation | JOA  Cervical ROM |
| PT, physiotherapy; OT, occupational therapy; mJOA, modified Japanese Orthopaedic Association; NDI, Neck Disability Index; SF-36 PCS, Short-Form 36 Physical Component Summary; AMS, American Motor Score; SCIM-III, Spinal Cord Independence Measure third version; dSCIM-III, improvement in performance during rehab; SCI-ARMI, Spinal Cord Ability Realization Measurement Index; VAS, Visual Analogue Scale; JOACMEQ, Japanese orthopaedic association cervical myelopathy evaluation questionnaire; ROM, range of motion; JOA, Japanese Orthopedic Association; EQ-5D-5L, EuroQoL 5-dimension 5-level; SER, MA, multidisciplinary approach; SC, standard care; Self-efficacy rehabilitation; DHPM, diversified health promoting model; CHPM, conventional health promoting model; PT+rTMS, physiotherapy plus repetitive transcranial magnetic stimulation; UEMS, upper extremity motor score; LEMS, lower extremity motor score; PBT, pertubation-based balance training; JOACMEQ-LEF, Japanese Orthopaedic Association Cervical Myelopathy Evaluation Questionnaire - Lower extremity function; TIN, timeliness incentive nursing; RN, routine nursing. | | | | | | | |

**Table S3.** Description of Post-operative Rehabilitation Programs

| **Reference** | **Initiation of Physical Therapy Intervention** | **Duration of Treatment** | **Frequency of Treatment** | **Treatment** |
| --- | --- | --- | --- | --- |
| Rahman et al. 2024 | - Inpatient PT/OT: Immediately post-op  - Outpatient OT/PT: Early (<42 days) or delayed (>42 days) | - Inpatient rehabilitation: During hospital stay (~3.7 days); Outpatient rehabilitation: Up to 12 months | - Inpatient: Daily therapy  - Outpatient: Varies (median 6 PT sessions, range 2-22) | - Inpatient OT/PT: Recovery from surgery, discharge planning  - Outpatient OT/PT: Neurological recovery, cervical ROM/stabilization, gait training, upper limb mobility |
| Catz et al. 2024 | - Inpatient rehabilitation initiated after spinal surgery | - Mean rehabilitation duration: 72 days (SD = 40) | - Daily inpatient multidisciplinary rehabilitation | - Comprehensive inpatient rehabilitation conducted in a spinal cord lesion (SCL) unit managed by a multidisciplinary team including physiatrists, nurses, physiotherapists, occupational therapists, social workers, psychologists.  - Medical and nursing interventions: Preventing and managing complications.  - Physical and occupational therapy: Focus on strength, ROM, and function using manual and other techniques.  - Psychosocial support: Psychological and social work interventions to aid coping with disability and community reintegration. |
| Uehara et al. 2022 | - Postoperative Day 2  - Subacute rehabilitation (2 weeks inpatient acute care followed by 2-3 weeks in a recovery-phase rehabilitation hospital before discharge home) | - 3 months | - Daily supervised physiotherapy during hospitalization (2 weeks)  - Self-directed home-based program post-discharge | **Intervention Group (Exercise Group):**  - Neck extension isometric muscle strengthening.  - Cervical ROM exercises.  - Stretching exercises (i.e., cervical paraspinal, UFT, SCM, scalenes)  - Scapular mobility exercises.  - Postural correction training using mirror feedback.  - Self-exercise continuation post-discharge.  **Control Group:**  - No structured neck exercises; general movement allowed within pain tolerance  - Postural correction guidance without active exercises. |
| Tamai et al. 2022 | - Postoperative Day 1: Patients begin sitting with a soft neck collar, standing, and walking. Brace removed after 1 week  - Outpatient rehabilitation once per week for 6 months postoperatively (40 minutes of PT and OT per session). | - Up to 6 months of postoperative rehabilitation | - SC Group: 40 min of PT/OT once per week for 6 months.  - MA Group: 40 min of PT/OT once per week + additional structured interventions for social functioning (SF). | **SC Rehabilitation:**  - Resistance training for atrophied upper limb muscles.  - Fine finger movement exercises.  - Daily activity training.  - Postural control training.  - Coordination exercises for lower limbs.  - Rhythmic movement exercises using an ergometer/treadmill.  **MA Rehabilitation:**  - Structured team (spine surgeons, physiotherapists, occupational therapists, clinical psychologists, nurses, social workers).  - Preoperative psychological evaluation.  - Personalized rehabilitation plans.  - Regular meetings to assess progress and adjust interventions.  - Targeted social reintegration support. |
| Lin et al. 2024 | - Control Group: CHPM initiated at hospital admission.  - Intervention Group: DHPM initiated preoperatively with additional guidance.  - Inpatient rehabilitation | - Hospital stay (~ 8 days) - Rehabilitation exercises continued post-discharge | - CHPM: Standard rehabilitation guidance at key time points.  - DHPM: Daily supervised rehabilitation sessions led by nurses, plus home-based training. | **CHPM:**  - Standard preoperative patient education.  - Basic postoperative rehabilitation guidance.  - General instructions on movement restrictions and ADLs.  **DHPM:**  Prehabilitation Phase:  - Collar support education.  - Pelvic floor muscle training.  - Venous thromboembolism prevention.  - Breathing exercises  - Positional adaptation training.  Postoperative Early Mobilization (Day 1-3):  - Day 1: Guided bed mobility (rolling, sitting), independent eating, self-care in bed.  - Day 2: Assisted transition from sitting to standing, standing balance training.  - Day 3: Supervised indoor walking (twice daily, 20 min per session), ADLs (grooming, bathing, toileting).  Muscle Strengthening and Mobility Training:  - Neck and shoulder relaxation exercises.  - Upper-limb training machine (30 min, twice daily).  Educational Support:  - Schematic diagrams for rehabilitation exercises.  - Video rehabilitation guidance (daily sessions on TV).  Assistive Devices:  - Custom-designed neck immobilizer for stabilization.  - Adjustable device to reduce post-op discomfort. |
| Farrokhi et al. 2024 | - Outpatient postoperative PT initiated within 6 months after decompression surgery.  - PT+ rTMS group began stimulation sessions immediately post-randomization. | - 2 weeks of structured therapy. | - 5 sessions per week.  - PT+ rTMS Group: 15 minutes of rTMS followed by 30 minutes of PT. - PT Group: 30 minutes of PT alone. | **PT+ rTMS Group:**  - rTMS: High-frequency (10 Hz) stimulation of the motor cortex. 1800 pulses per session (900 to left motor cortex, 900 to right motor cortex).  - Strengthening and stretching exercises for the neck, shoulder, and upper limbs.  - Walking and weight transfer exercises.  - ROM exercises for upper and lower extremities.  **PT Group:**  - Same PT protocol as above without cortical stimulation. |
| Cheung et al. 2019 | - Postoperative Day 1.  - Collar Group: Required to wear a Philadelphia collar for 3 weeks postoperatively.  - No-Collar Group: Allowed free mobilization immediately after surgery. | - Follow-up assessments conducted at 1, 2, 3, and 6 weeks, and at 3, 6, and 12 months postoperatively. | - Collar Group: Wore a rigid cervical collar continuously for 3 weeks, except while resting in bed.  - No-Collar Group: Encouraged unrestricted neck movements postoperatively. | **General Postoperative Rehabilitation Protocol:**  - Respiratory and circulatory exercises.  - Transfer and walking training.  - ADLs.  - General home exercise program (postural training, walking).  **Collar Group:**  - Required to wear a rigid cervical collar for 3 weeks postoperatively.  - Collar removed temporarily while in bed.  **No-Collar Group:**  - Allowed unrestricted neck motion postoperatively. |
| Cheng et al. 2020 | Outpatient postoperative rehabilitation initiated at least 6 months after decompression surgery.  - Baseline balance assessments conducted before training. | - 4-week intervention program. | - Two 1-hour sessions per week (8 sessions total).  - Each session consisted of 80 perturbations (20 forward, 20 backward, 20 right-to-left, 20 left-to-right). | **PBT Protocol:**  - Conducted on a split-belt perturbation treadmill.  - Participants wore a safety suspension harness.  - Standing perturbation training: Forward perturbations at 0.15-0.20 m/s, backward perturbations at 0.20-0.25 m/s, lateral perturbations at 0.09-0.18 m/s.  - Walking training: treadmill speed set at 0.8× comfortable walking speed, speed increased by 0.05 m/s each session. |
| Yue & Liu 2021 | - RN: Postoperative day 1.  - TIN: Postoperative day 1, with structured psychological and motivational support.  - Inpatient rehabilitation, transitioned to outpatient follow-up visits. | - 2-month rehabilitation period. | - RN: Standard postoperative nursing care.  - TIN: motivational interventions, guided rehabilitation, phone call follow-ups for non-compliant patients. | **RN:**  - Bed rest with cervical collar for 1-10 days.  - Passive and active limb exercises starting postop day 2  - Assisted sitting and standing training from postop day 5.  - Walking with support on postop day 11.  - Cervical collar removed at postop day 20 for gentle neck movement training.  - Full neck training without collar after 1-month postop.  **TIN:**  - Spiritual Incentive: Nurses provided encouragement to reinforce behavior and motivation.  - Goal Incentive: Patients were given specific targets (e.g., joint mobility exercises 15 min, 2-3 times/day).  - Model Incentive: Successful patient cases were shared as role models.  - Benefit Incentive: Education on the benefits of rehabilitation compliance.  - Post-discharge monitoring  - Phone call follow-ups for non-compliant patients. |
| Iizuka et al. 2005 | -Postoperative Day 1: Cervical collar applied immediately after surgery. | - Group 8W: Wore a cervical collar for 8 weeks postoperatively.  - Group 4W: Wore a cervical collar for 4 weeks postoperatively.  - Follow-up duration: Mean 27-32 months. | - Collar usage: Continuous during designated period except while lying down.  - No structured PT provided. | **Group 8W (8-Week Collar Use):**  - Wore a rigid cervical collar for 8 weeks postoperatively.  - Allowed gradual cervical mobility exercises after collar removal. **Group 4W (4-Week Collar Use):**  - Wore a rigid cervical collar for 4 weeks postoperatively.  - Allowed gradual cervical mobility exercises after collar removal. |
| PT, physiotherapy; OT, occupational therapy; ROM, range of motion; UFT, upper fibers of the trapezius; SCM, sternocleidomastoid; SC, standard care; MA, multidisciplinary approach; DHPM, diversified health promoting model; CHPM, conventional health promoting model; ADL, activities of daily living; PT+rTMS, physiotherapy plus repetitive transcranial magnetic stimulation; PBT, pertubation-based balance training; VAS, Visual Analogue Scale; TUG, Timed Up and Go; TIN, timeliness incentive nursing; RN, routine nursing; postop, postoperative. | | | | |

**Table S4.** Outcomes of Included Studies

| **Reference** | **Follow-up** | **Intervention** | **Findings - Research Group** | **Findings - Control Group** |
| --- | --- | --- | --- | --- |
| Rahman et al. 2024 | Pre-operative  12-month post-operative | OT  PT | **mJOA:**  Preop score: 13.1 (SD ± 2.3)  Postop scores at 12 months: 14.2 (SD ± 2.4)  Mean improvement: +1.1 (SD ± 2.6)  **Multivariate Regression Results (Impact of Rehabilitation on mJOA):**  Preop score: Beta = -0.65, 95% CI = -0.89 to -0.42, p<0.001  OT: Beta =1.6, 95% CI 0.28 to 3.0, p-value = 0.019  PT: Beta = -0.32, 95% CI = -1.5 to 0.9, p = 0.6  Timing of Therapy (>42 days vs. <42 days): Beta = -0.9, 95% CI = -2.7 to 0.8, p = 0.26  Type of Therapy (Gait/Hand Function Training vs. Cervical ROM/Mobilization): -Beta = -2.2, 95% CI = -3.9 to -0.4, p = 0.02  Number of Therapy Sessions: Beta = 0.05, 95% CI = -0.08 to 0.2, p = 0.42​  **SF-36 PCS:**  Preop score: 36 (SD ±10)  Postop scores at 12 months: 42.3 (SD ±11)  Mean improvement: -9.7 (SD ± 17.9)  **Multivariate Regression Results (Impact of Rehabilitation on SF-36 PCS Score):**  OT: Beta = 7.7, 95% CI = 2.0 to 13, p = 0.009  PT: Beta = -1.6, 95% CI = -6.7 to 3.5, p = 0.5  Timing of Therapy: Beta = -7.6, 95% CI = -14.6 to -0.7, p = 0.03  Type of Therapy: Beta = -7.6, 95% CI = -14.7 to -0.5, p = 0.04  Number of Therapy Sessions: Beta = -0.68, 95% CI = -1.2 to -0.16, p = 0.01​  **NDI Score**  Preop score: 35 (SD ± 19)  Postop scores at 12 months: 24.9 (SD ± 18.5)  Mean improvement: -6.3 (SD ± 10.5)  **Multivariate Regression Results (Impact of Rehabilitation on NDI Score):**  OT: Beta = -6.37, 95% CI = -17 to 4.1, p = 0.2  PT: Beta = 3.38, 95% CI = -5.9 to 13, p = 0.5  Timing of Therapy: Beta = 1.6, 95% CI = -11.3 to 14.5, p = 0.8  Type of Therapy: Beta = 12.8, 95% CI = -0.5 to 25.9, p = 0.06  Number of Therapy Sessions: Beta = 0.5, 95% CI = -0.4 to 1.5, p = 0.3​ |  |
| Catz et al. 2024 | Admission (t=0)  Discharge (t=72 days) | (1) Comprehensive rehabilitation group | **1. AMS:**  Admission AMS: 74.9 (SD = 19.8)  Discharge AMS: 85.0 (SD = 15.3)  Mean improvement: +10.1 (SD = 12.7), p < 0.001  **2. SCIM-III:**  Admission SCIM-III: 49.5 (SD = 23.4)  Discharge SCIM-III: 71.5 (SD = 20.5)  Mean improvement: +22.0 (SD = 17.8), p < 0.001​  **3. SCIM-95:**  Admission SCIM-95: 84.3 (SD = 15.0)  Discharge SCIM-95: 90.5 (SD = 10.8)  Mean change: +6.2  **4. SCI-ARMI:**  Admission SCI-ARMI: 57.3 (SD = 24.1)  Discharge SCI-ARMI: 77.9 (SD = 19.2)  Mean change: +20.5 (SD = 19.4), p < 0.001​. |  |
| Uehara et al. 2022 | Baseline  2-week follow-up  3-month follow-up | (1) Cervical exercise group  (2) Control group | **Primary Outcome:**  **VAS:**  Exercise Group  Preoperative: 27.1 (SD = 26.6)  2 Weeks Postoperative: 42.8 (SD = 24.0)  3 Months Postoperative: 25.3 (SD = 23.1)  ANOVA:  Effect of Group: F = 0.259, p=0.613  Effect of Time: F = 18.328, p < 0.001  Group × Time Interaction: F = 0.476, p = 0.623  **JOACMEQ - Cervical Spine Function**  Exercise Group  Preoperative: 71.8 (SD = 21.9)  2 Weeks Postoperative: –19.2 (SD = 30.8)  3 Months Postoperative: 3.2 (SD = 19.9)  **Secondary Outcomes:**  **Neck Extension Muscle Strength (N/kg)**  Exercise Group  Preoperative: 1.62 (SD = 0.56)  2 Weeks Postoperative: 1.31 (SD = 0.43)  3 Months Postoperative: 1.69 (SD = 0.53)  ANOVA:  Effect of Group: F = 0.092, p=0.763  Effect of Time: F = 26.550, p < 0.001  Group × Time Interaction: F = 0.304, p = 0.738  **Neck Flexion Muscle Strength (N/kg)**  Exercise Group  Preoperative: 1.26 (SD = 0.34)  2 Weeks Postoperative: 1.23 (SD = 0.40)  3 Months Postoperative: 1.41 (SD = 0.33)  ANOVA:  Effect of group: F = 0.606, p=0.440  Effect of Time: F = 18.201, p < 0.001  Group × Time Interaction: F = 2.747, p = 0.068  **C2–7 lordosis (deg.)**  Exercise Group  Preoperative: 12.0±9.9  2 Weeks Postoperative: N/A  3 Months Postoperative: 8.7±13.1  ANOVA:  Effect of group: F = 0.301, p = 0.586  Effect of Time: F = 12.085, p = 0.001  Group × Time Interaction: F = 0.224, p = 0.638  **C7 Slope (degrees)**  Exercise Group  Preoperative: 27.7° (SD = 8.6)  3 Months Postoperative: 26.0° (SD = 6.4)  ANOVA:  Effect of group: F = 1.330, p = 0.253  Effect of Time: F = 5.454, p = 0.023  Group × Time Interaction: F = 0.092, p = 0.763  **C2-C7 SVA (mm)**  Exercise Group  Preoperative: 29.3 mm (SD = 17.8)  3 Months Postoperative: 30.3 mm (SD = 18.5)  ANOVA:  Effect of group: F = 3.645, p = 0.061  Effect of Time: F = 0.494, p = 0.485  Group × Time Interaction: F = 0.004, p = 0.949  **Cervical ROM**  Exercise Group  Preoperative: 41.7° (SD = 10.9)  3 Months Postoperative: 30.0° (SD = 10.9)  ANOVA:  Effect of group: F = 0.250, p = 0.619  Effect of Time: F = 61.160, p < 0.001  Group × Time Interaction: F = 0.086, p = 0.771  **Right Bending (degrees)**  Exercise Group  Preoperative: 23.7° (SD = 6.9)  3 Months Postoperative: 25.8° (SD = 7.0)  ANOVA:  Effect of group: F = 0.760, p = 0.387  Effect of Time: F = 4.966, p = 0.030  Group × Time Interaction: F = 0.332, p = 0.567  **Left Bending (degrees)**  Exercise Group  Preoperative: 23.9° (SD = 8.0)  3 Months Postoperative: 25.3° (SD = 9.0)  ANOVA:  Effect of group: F = 0.014, p = 0.906  Effect of Time: F = 5.979, p = 0.018  Group × Time Interaction: F = 0.886, p = 0.350  **Right Rotation (degrees)**  Exercise Group  Preoperative: 56.8° (SD = 13.0)  3 Months Postoperative: 60.0° (SD = 8.1)  ANOVA:  Effect of group: F = 0.244, p = 0.623  Effect of Time: F = 6.777, p = 0.012  Group x Time Interaction: F = 0.000, p = 0.996  **Left Rotation (degrees)**  Exercise Group  Preoperative: 56.7° (SD = 12.7)  3 Months Postoperative: 60.4° (SD = 9.4)  ANOVA:  Effect of group: F = 0.030, p = 0.862  Effect of Time: F = 7.374, p = 0.009  Group x Time Interaction: F = 0.074, p = 0.786 | **Primary Outcome:**  **VAS:**  Control Group  Preoperative: 23.1 (SD = 22.7)  2 Weeks Postoperative: 44.9 (SD = 27.4)  3 Months Postoperative: 20.6 (SD = 18.3)  **JOACMEQ - Cervical Spine Function**  Control Group  Preoperative: 75.1 (SD = 22.5)  2 Weeks Postoperative: –12.0 (SD = 25.0)  3 Months Postoperative: 7.4 (SD = 26.5)  **Secondary Outcomes:**  **Neck Extension Muscle Strength (N/kg)**  Control Group  Preoperative: 1.61 (SD = 0.47)  2 Weeks Postoperative: 1.30 (SD = 0.39)  3 Months Postoperative: 1.61 (SD = 0.47)  **Neck Flexion Muscle Strength (N/kg)**  Control Group  Preoperative: 1.41 (SD = 0.33)  2 Weeks Postoperative: 1.25 (SD = 0.36)  3 Months Postoperative: 1.44 (SD = 0.36)  **C2–7 lordosis (deg.)**  Control group:  Preoperative: 14.0±11.0  2 Weeks Postoperative: N/A  3 Months Postoperative: 9.6±10.9  **C7 Slope (degrees)**  Control Group  Preoperative: 25.4° (SD = 7.7)  3 Months Postoperative: 24.1° (SD = 8.6)  **C2-C7 SVA (mm)**  Control Group  Preoperative: 21.7 mm (SD = 14.8)  3 Months Postoperative: 22.6 mm (SD = 13.1)  **Cervical ROM**  Control Group  Preoperative: 41.0° (SD = 12.8)  3 Months Postoperative: 28.3° (SD = 9.8)  **Right Bending (degrees)**  Control Group  Preoperative: 25.6° (SD = 8.0)  3 Months Postoperative: 26.9° (SD = 7.0)  **Left Bending (degrees)**  Control Group  Preoperative: 22.7° (SD = 8.0)  3 Months Postoperative: 26.0° (SD = 7.6)  **Right Rotation (degrees)**  Control Group  Preoperative: 55.7° (SD = 9.8)  3 Months Postoperative: 58.9° (SD = 5.8)  **Left Rotation (degrees)**  Control Group  Preoperative: 56.6° (SD = 10.0)  3 Months Postoperative: 59.6° (SD = 7.5) |
| Tamai et al. 2022 | Pre-operative  1-year post-operative | (1) Multi-disciplinary protocol group  (2) Standard care group | **1. JOA Score (points)**  **MA Cohort Total Score:**  Preoperatively: 9.6 ± 2.5  1 year postoperatively: 14.7 ± 1.5  p=0.040  **Upper Limb Function:**  Preoperatively: 1.94 ± 0.91  1 year postoperatively: 3.65 ± 0.48  p=0.033  **Lower Limb Function:**  Preoperatively: 1.82 ± 0.89  1 year postoperatively: 3.06 ± 0.99  p=0.256  **Upper Limb Sensory:**  Preoperatively: 0.85 ± 0.41  1 year postoperatively: 1.45 ± 0.37  p=0.476  **Lower Limb Sensory:**  Preoperatively: 1.37 ± 0.49  1 year postoperatively: 1.74 ± 0.33  p=0.593  **2. EQ-5D-5L Scores**  **Morbidity**  Preoperative: -0.10 ± 0.08, p=0.991  1-Year Post-op: -0.07 ± 0.06, p =0.862  **Self-care**  Preoperative: -0.05 ± 0.06, p= 0.197  1-Year Post-op: -0.02 ± 0.04, p=0.047  **Activities**  Preoperative: -0.08 ± 0.06, p=0.763  1-Year Post-op: -0.04 ± 0.04, p=0.058  **Pain**  Preoperative: -0.09 ± 0.05, p=0.378  1-Year Post-op: -0.04 ± 0.03, p=0.117  **Anxiety/Depression**  Preoperative: -0.06 ± 0.06, p=0.498  1-Year Post-op: -0.04 ± 0.05, p= 0.964  **Total Score**  Preoperative: 0.62 ± 0.23, p=0.690  1-Year Post-op: 0.79 ± 0.17, p=0.145  **3. NDI (%)**  Preoperative: 31.5 ± 20.3, p=0.690  1-Year Post-op: 18.6 ± 13.1, p=0.342  **4. VAS Pain Scores (mm)**  **Neck Pain**  Preoperative: 27.1 ± 32.7, p=0.635  1-Year Post-op: 16.2 ± 21.2, p= 0.635  **Arm Pain**  Preoperative: 52.9 ± 36.9, p= 0.138  1-Year Post-op: 28.6 ± 32.3, p= 0.297  **Arm Numbness**  Preoperative: 63.8 ± 31.3, p=0.669  1-Year Post-op: 33.6 ± 33.0, p=0.462  **5. JOACMEQ Scores**  **Cervical Function**  Preoperative: 63.2 ± 29.3, p=0.751  1-Year Post-op: 78.6 ± 20.8, p=0.118  **Upper Extremity Function**  Preoperative: 71.3 ± 26.4, p=0.321  1-Year Post-op: 88.0 ± 12.3, p=0.001  **Lower Extremity Function**  Preoperative: 54.7 ± 28.9, p=0.463  1-Year Post-op: 69.1 ± 57.6, p=0.060  **QoL**  Preoperative: 45.1 ± 17.7, p=0.609  1-Year Post-op: 54.2 ± 16.9, p=0.374 | **1. JOA Score**  **Control Total Score:**  Preoperatively: 9.3 ± 2.5  1 year postoperatively: 13.2 ± 2.2  **Upper Limb Function:**  Preoperatively: 1.92 ± 0.97  1 year postoperatively: 3.13 ± 0.74  **Lower Limb Function:**  Preoperatively: 1.53 ± 0.96  1 year postoperatively: 2.55 ± 0.98  **Upper Limb Sensory:**  Preoperatively: 0.93 ± 0.35  1 year postoperatively: 1.38 ± 0.32  **Lower Limb Sensory:**  Preoperatively: 1.30 ± 0.53  1 year postoperatively: 1.69 ± 0.36  **2. EQ-5D-5L Scores**  **Morbidity**  Preoperative: -0.11 ± 0.08  1-Year Post-op: -0.09 ± 0.07  **Self-care**  Preoperative: -0.07 ± 0.06  1-Year Post-op: -0.04 ± 0.05  **Activities**  Preoperative: -0.08 ± 0.05  1-Year Post-op: -0.06 ± 0.05  **Pain**  Preoperative: -0.08 ± 0.06  1-Year Post-op: -0.04 ± 0.05  **Anxiety/Depression**  Preoperative: -0.08 ± 0.06  1-Year Post-op: -0.04 ± 0.05  **Total Score**  Preoperative: 0.59 ± 0.24  1-Year Post-op: 0.72 ± 0.21  **3. NDI (%)**  Preoperative: 31.3 ± 18.4  1-Year Post-op: 21.0 ± 15.3  **4. VAS Pain Scores (mm)**  **Neck Pain**  Preoperative: 30.8 ± 31.7  1-Year Post-op: 15.7 ± 21.7  **Arm Pain**  Preoperative: 43.5 ± 36.9  1-Year Post-op: 20.0 ± 24.7  **Arm Numbness**  Preoperative: 62.7 ± 30.6  1-Year Post-op: 38.0 ± 30.6  **5. JOACMEQ Scores**  **Cervical Function**  Preoperative: 58.6 ± 29.8  1-Year Post-op: 71.5 ± 25.6  **Upper Extremity Function**  Preoperative: 63.5 ± 24.2  1-Year Post-op: 74.9 ± 20.6  **Lower Extremity Function**  Preoperative: 48.9 ± 29.9  1-Year Post-op: 57.6 ± 25.5  **QoL**  Preoperative: 42.4 ± 18.7  1-Year Post-op: 50.9 ± 18.4 |
| Lin et al. 2024 | Pre-intervention  Day 3 post-operative  Prior to discharge | (1) Cervical exercise group  (2) Control group | **1. SER Outcome**  Pre-Int: 65.40 ± 10.15  3 Days Post: 73.58 ± 8.87  Prior to Discharge: 81.82 ± 6.95  No significant difference between groups at baseline (p=0.980).  Significant difference between groups 3 days post-intervention (p=0.040).  Significant difference between groups prior to discharge (p<0.001).  **2. Grip Strength of Affected Limb**  Intervention Group:  Pre-Int: 17.97 ± 6.84  3 Days Post: 16.89 ± 6.31  Prior to Discharge: 21.05 ± 6.79  No significant difference between groups at baseline (p=788).  No significant difference between groups 3 days post-intervention (p=0.998).  Significant difference between groups prior to discharge (p=0.041).  **3. JOA Score**  Pre-Int: 11.91 ± 1.88  3 Days Post: 11.53 ± 1.39  Prior to Discharge: 13.64 ± 1.22  No significant difference between groups at baseline (p=0.703).  No significant difference between groups 3 days post-intervention (p=0.975).  Significant difference between groups prior to discharge (p=0.001).  **4. Barthel Index**  Pre-Int: 87.27 ± 8.54  3 Days Post: 80.82 ± 6.86  Prior to Discharge: 94.27 ± 4.56  No significant difference between groups at baseline (p=0.661).  No significant difference between groups 3 days post-intervention (p=0.753).  Significant difference between groups prior to discharge (p=0.003). | **1. SER Outcome**  Pre-Int: 65.44 ± 6.64  3 Days Post: 70.25 ± 7.62  Prior to Discharge: 75.33 ± 7.66  **2. Grip Strength of Affected Limb**  Pre-Int: 17.61 ± 7.04  3 Days Post: 16.89 ± 6.56  Prior to Discharge: 18.38 ± 6.49  **3. JOA Score**  Pre-Int: 11.77 ± 1.91  3 Days Post: 11.52 ± 1.24  Prior to Discharge: 12.83 ± 1.25  **4. Barthel Index**  Pre-Int: 86.54 ± 8.72  3 Days Post: 80.38 ± 7.33  Prior to Discharge: 91.44 ± 5.08 |
| Farrokhi et al. 2024 | Before intervention    After intervention | (1) PT + rTMS group  (2) PT only group | **1. RTMS**  PT + rTMS:  Pre-Int: 42.23 ± 6.06  Post-Rehabilitation: 44.61 ± 4.94  p=0.000  **2. LTMS**  Pre-Int: 42.31 ± 6.29  Post-Rehabilitation: 44.92 ± 4.73  p=0.003  **3. UEMS**  Pre-Int: 40.39 ± 6.71  Post-Rehabilitation: 44.31 ± 4.85  p=0.000  **4. LEMS**  Pre-Int: 44.08 ± 5.89  Post-Rehabilitation: 45.23 ± 5.00  p=0.004  **5. mJOA Scale**  Pre-Int: 12.38 ± 3.17  Post-Rehabilitation: 14.38 ± 2.46  p=0.000  **6. Ashworth Scale**  Pre-Int: 1.77 ± 0.60  Post-Rehabilitation: 0.85 ± 0.55  p=0.003  **7. Nurick Grade**  Pre-Int: 2.31 ± 0.96  Post-Rehabilitation: 1.54 ± 0.94  p=0.001 | **1. RTMS**  PT only:  Pre-Int: 44.00 ± 8.52  Post-Rehabilitation: 44.85 ± 7.41  p=0.003  **2. LTMS**  Pre-Int: 43.08 ± 8.27  Post-Rehabilitation: 44.15 ± 7.14  p= 0.001  **3. UEMS**  Pre-Int: 43.15 ± 8.36  Post-Rehabilitation: 43.92 ± 7.53  p= 0.005  **4. LEMS**  Pre-Int: 43.77 ± 8.41  Post-Rehabilitation: 45.08 ± 7.05  p< 0.001  **5. mJOA Scale**  Pre-Int: 14.53 ± 2.93  Post-Rehabilitation: 15.00 ± 2.50  p=0.001  **6. Ashworth Scale**  Pre-Int: 1.54 ± 0.77  Post-Rehabilitation: 1.23 ± 0.43  p=0.003  **7. Nurick Grade**  Pre-Int: 2.46 ± 0.87  Post-Rehabilitation: 2.07 ± 0.64  p=0.001 |
| Cheung et al. 2019 | Preoperatively    Postoperative 1, 2, 3, and 6 wk, and 3, 6, and 12 months | (1) Cervical collar group  (2) No cervical collar group | **1. VAS for Axial Neck Pain**  Preoperative: Collar: 2.7 ± 2.7; No Collar: 2.1 ± 1.9, p=0.659  Postoperative 1-wk: Collar: 3.5 ± 2.0; No Collar: 5.4 ± 2.5, p=0.038  Postoperative 2-wk: Collar: 1.5 ± 1.4; No Collar: 3.5 ± 2.4, p=0.028  Postoperative 3-wk: Collar: 1.3 ± 1.0; No Collar: 2.8 ± 1.9, p=0.031  Postoperative 6-wk: Collar: 0.9 ± 0.9; No Collar: 1.9 ± 1.7, p=0.139  Postoperative 3-mo: Collar: 1.4 ± 1.6; No Collar: 1.6 ± 1.8, p=0.873  Postoperative 6-mo: Collar: 1.3 ± 1.8; No Collar: 1.3 ± 1.5, p=0.811  Postoperative 12-mo: Collar: 1.1 ± 1.7; No Collar: 1.7 ± 2.4, p=0.607  **2. NDI (%; Mean ± SD; P-Values)**  Preoperative: Collar: 31.8 ± 16.9; No Collar: 31.0 ± 11.0, p=0.931  Postoperative 1-wk: Collar: 34.2 ± 17.7; No Collar: 44.5 ± 11.2, p=0.252  Postoperative 2-wk: Collar: 32.0 ± 15.9; No Collar: 47.0 ± 14.4, p=0.094  Postoperative 3-wk: Collar: 28.4 ± 19.5; No Collar: 33.1 ± 14.0, p=0.573  Postoperative 6-wk: Collar: 24.8 ± 10.0; No Collar: 34.0 ± 9.5, p=0.147  Postoperative 3-mo: Collar: 22.5 ± 18.1; No Collar: 22.8 ± 7.7, p=0.796  Postoperative 6-mo: Collar: 22.4 ± 18.3; No Collar: 23.1 ± 12.5, p=0.608  Postoperative 12-mo: Collar: 20.8 ± 18.0; No Collar: 22.9 ± 10.7, p=0.695  **3. mJOA Score**  Preoperative: Collar: 9.8 ± 4.0; No Collar: 10.7 ± 3.0, p=0.610  Postoperative 1-wk: Collar: 11.7 ± 2.6; No Collar: 12.1 ± 2.9, p=0.706  Postoperative 2-wk: Collar: 11.9 ± 3.2; No Collar: 12.3 ± 2.2, p=0.970  Postoperative 3-wk: Collar: 12.5 ± 2.7; No Collar: 12.5 ± 2.2, p=0.794  Postoperative 6-wk: Collar: 13.8 ± 1.5; No Collar: 13.3 ± 1.9, p=0.613  Postoperative 3-mo: Collar: 14.0 ± 1.4; No Collar: 13.8 ± 1.6, p=0.798  Postoperative 6-mo: Collar: 13.8 ± 1.3; No Collar: 14.0 ± 2.0, p=1.000  Postoperative 12-mo: Collar: 14.1 ± 1.4; No Collar: 14.5 ± 1.8, p=0.636  **SF-36 Subscales**  **1. Physical Functioning**  Preoperative: Collar: 32.7 ± 21.8; No Collar: 43.3 ± 32.4  Post-operative 1-wk: Collar: 26.3 ± 26.5; No Collar: 28.8 ± 22.7  Postoperative 2-wk: Collar: 37.5 ± 33.4; No Collar: 32.6 ± 23.7  Postoperative 12-mo: Collar: 64.6 ± 18.4; No Collar: 50.6 ± 25.4, p<0.05  **2. Role Limitations Due to Physical Health**  Preoperative: Collar: 6.8 ± 16.2; No Collar: 12.5 ± 31.1  Post-operative 1-wk: Collar: 13.3 ± 28.1; No collar: 13.2 ± 33.2  Postoperative 12-mo: Collar: 25.0 ± 40.8; No Collar: 32.4 ± 41.2, p>0.05  **3. Role Limitations Due to Emotional Problems**  Preoperative: Collar: 36.4 ± 45.8; No Collar: 47.2 ± 50.2  Post-operative 1-wk: Collar: 26.7 ± 38.2; No collar: 27.5 ± 41.2, p>0.05  Postoperative 12-mo: Collar: 43.6 ± 45.9; No Collar: 52.9 ± 47.2, p>0.05  **4. Vitality**  Preoperative: Collar: 39.5 ± 21.7; No Collar: 38.3 ± 24.2  Post-operative 1-wk: Collar: 48.7 ± 16.6; No collar: 44.1 ± 15.0,  Postoperative 12-mo: Collar: 49.2 ± 27.5; No Collar: 56.2 ± 16.3  **5. Mental Health**  Preoperative: Collar: 54.5 ± 28.7; No Collar: 67.7 ± 20.7  C Collar: 58.7 ± 14.5; No collar: 57.2 ± 18.4  Postoperative 12-mo: Collar: 74.8 ± 20.7; No Collar: 70.8 ± 16.5  **6. Social Functioning**  Preoperative: Collar: 40.9 ± 30.2; No Collar: 52.1 ± 26.0  Post-operative 1-wk: Collar: 50.0 ± 32.0; No collar: 47.8 ± 30.1  Postoperative 12-mo: Collar: 76.9 ± 19.7; No Collar: 66.9 ± 26.1  **7. Bodily Pain**  Preoperative: Collar: 42.2 ± 26.2; No Collar: 47.1 ± 22.1  Post-operative 1-wk: Collar: 44.9 ± 17.2; No collar: 34.9 ± 19.0  Postoperative 12-mo: Collar: 61.4 ± 32.9; No Collar: 62.8 ± 24.5  **8. General Health Perception**  Preoperative: Collar: 39.6 ± 16.4; No Collar: 43.3 ± 20.8  Post-operative 1-wk: Collar: 48.1 ± 18.6; No collar: 43.6 ± 16.3  Postoperative 12-mo: Collar: 50.9 ± 25.4; No Collar: 53.0 ± 17.9  **9. Physical Component Summary (PCS)**  Preoperative: Collar: 28.8 ± 5.8; No Collar: 30.4 ± 9.5  Post-operative 1-wk: Collar: 29.6 ± 7.9; No collar: 28.3 ± 6.4  Postoperative 12-mo: Collar: 39.0 ± 9.8; No Collar: 36.7 ± 9.2  **10. Mental Component Summary (MCS)**  Preoperative: Collar: 41.7 ± 11.7; No Collar: 46.1 ± 11.4  Post-operative 1-wk: Collar: 43.4 ± 8.4; No collar: 42.9 ± 8.7  Postoperative 12-mo: Collar: 48.3 ± 11.2; No Collar: 48.9 ± 9.0  **Cervical ROM Data**  **1. Extension (Degrees)**  Preoperative: Collar: 43.9 ± 13.6; No Collar: 51.0 ± 54.5, p= 0.095  Post-operative 1-wk: Collar: 34.4 ± 14.8; No collar: 32.9 ± 15.9, p=0.832  Postoperative 12-mo: Collar: 59.6 ± 12.7; No Collar: 57.7 ± 14.8, p= 0.683  **2. Flexion (Degrees)**  Preoperative: Collar: 55.5 ± 11.7; No Collar: 54.5 ± 18.0, p=0.909  Post-operative 1-wk: Collar: 24.5 ± 10.0; No collar: 26.3 ± 14.1, p=0.909  Postoperative 12-mo: Collar: 49.1 ± 11.3; No Collar: 51.9 ± 7.0, p=0.367  **3. Lateral Flexion - Right (Degrees)**  Preoperative: Collar: 35.4 ± 7.8; No Collar: 37.2 ± 13.4, p= 0.883  Post-operative 1-wk: Collar: 26.8 ± 10.8; No collar: 26.4 ± 11.6, p=0.806  Postoperative 12-mo: Collar: 37.2 ± 9.3; No Collar: 39.8 ± 9.3, p=0.286  **4. Lateral Flexion - Left (Degrees)**  Preoperative: Collar: 34.9 ± 10.9; No Collar: 35.9 ± 14.3, p= 0.832  Post-operative 1-wk: Collar: 22.7 ± 9.1; No collar: 25.9 ± 13.3, p= 0.481  Postoperative 12-mo: Collar: 34.0 ± 9.4; No Collar: 38.4 ± 9.7, p=0.193  **5. Rotation - Right (Degrees)**  Preoperative: Collar: 58.9 ± 15.4; No Collar: 59.3 ± 14.9, p=0.909  Post-operative 1-wk: Collar: 41.8 ± 14.0; No collar: 45.1 ± 17.7, p=0.523  Postoperative 12-mo: Collar: 59.4 ± 11.2; No Collar: 61.6 ± 11.6, p=0.781  **6. Rotation - Left (Degrees)**  Preoperative: Collar: 54.1 ± 14.5; No Collar: 59.6 ± 14.1, p=0.271  Post-operative 1-wk: Collar: 42.9 ± 15.7; No collar: 46.9 ± 18.0, p=0.403  Postoperative 12-mo: Collar: 64.4 ± 9.9; No Collar: 66.2 ± 10.8, p= 0.545 |  |
| Cheng et al. 2020 | Before intervention    After intervention | (1) DCM group receiving Pertubation-based Balance Training  (2) Healthy control group | **1. VAS for Pain**  DCM Group Pre-training: 2.4 ± 2.3  Post-training: p= 1.0  **2. NDI (%)**  Pre-training: 12.7 ± 8.3  Post-training: p= 0.30  **3. JOACMEQ-LEF**  Pre-training: 74.3 ± 21.8  Post-training: p = 0.05  **4. Center of Pressure Mean Velocity (Static Postural Control)**  Pre-training: Significantly higher than controls (p < 0.01)  Post-training: Improved significantly (p = 0.02, d = 0.84), not significantly different from Control Group (p = 0.34)  **5. Center of Mass Variance (Dynamic Postural Control)**  Pre-training Forward Perturbation: No significant difference between groups (p=1.00).  Pre-training Backward Perturbation: DCM had significantly worse stability than controls (p = 0.04).  Post-training Forward Perturbation: Improved (p = 0.03, d = 0.65).  Post-training Backward Perturbation: Improved (p< 0.01, d = 0.86)  Change from Pre-training to Post-training: No significant between-group difference in forward perturbation (p=0.11) and backward perturbation (p=0.34) after training.  **6. Reaction Time to Perturbation**  Pre-training: DCM Group had significantly longer reaction times than controls (p < 0.01 for both forward and backward perturbations).  Post-training Forward Perturbation Reaction Time: Improved significantly (p < 0.01, d = 0.86).  Post-training Backward Perturbation Reaction Time: Improved significantly (p < 0.01, d = 1.72).  No significant between-group differences identified at post-training (p = 0.20 forward, p = 0.34 backward).  **7. Gait Speed (m/s)**  Pre-training: Significantly slower than controls (p = 0.03).  Post-training: Improved significantly (p = 0.04, d = 0.19)  No significant between-group differences after training (p = 0.15)  **8. TUG**  Pre-training: Significantly slower than controls (p < 0.01).  Post-training: Improved significantly (p < 0.01, d = 1.18)  No significant between-group differences after training (p = 0.15). | **1. VAS for Pain**  Healthy Control Pre-training: Not measured  **2. NDI (%)**  Pre-training: Not measured  **3. JOACMEQ-LEF**  Pre-training: Not measured  **4. Center of Pressure Mean Velocity**  Pre-training: Lower than DCM group (p < 0.01)  Post-training: Not different from DCM Group (p = 0.34)  **5. Center of Mass Variance**  Pre-training Forward Perturbation: No difference between groups (p=1.00).  Post-training Forward Pertubation: No difference between groups (p =0.11)  Post-training Backward Perturbation: No difference between groups (p = 0.34) |
| Yue & Liu 2021 | Baseline  2 months post-intervention | (1) Timeliness Incentive nursing group  (2) Control group - routine nursing | **Primary Outcome: Postoperative Recovery (Activity Time & Hospital Stay)**  **Out-of-bed activity time:** Research group (timeliness incentive nursing): significantly shorter than control group (p<0.001).  **Length of hospital stay:** Research group: significantly shorter than control group (p<0.001).  **Secondary Outcomes: Pain, Function, and Quality of Life**  **1. VAS**  Before intervention: No significant difference between groups (p>0.05).  After intervention (2 months post-op): VAS scores significantly decreased in both groups (p<0.001).  Greater pain reduction in the research group compared to the control group (p<0.001).  **2. JOA Score**  Before intervention: No significant difference between groups (p>0.05).  After intervention: Both groups improved significantly (p<0.001).  Research group had significantly better JOA scores than the control group (p<0.001).  **3. Cervical ROM (°)**  Before intervention: No significant difference between groups (p>0.05).  After intervention: ROM significantly increased in both groups (p<0.001).  Greater improvement in the research group compared to the control group (p<0.001).  **4. Complication Rates (%)**  Total complication rate: 3.85%, p=0.026 (difference between groups).  Lower incidence of wound edema, deep vein thrombosis, and spastic paralysis in the research group.  **5. SF-36 Score**  Physiological Function:  Before intervention: 52.07 ± 5.09, p=0.827  After intervention: 81.45 ± 8.51, p<0.001  Physical Pain:  Before intervention: 53.06 ± 7.38, p=0.370  After intervention:27.14 ± 7.84, p<0.001  Physiological Responsibility:  Before intervention: 74.53 ± 6.47, p=0.564  After intervention: 89.87 ± 8.28, p<0.001  Emotional Responsibility:  Before intervention: 62.34 ± 7.76, p=0.942  After intervention: 86.54 ± 9.21, p<0.001  Social Function:  Before intervention: 53.17 ± 6.86, p=0.580  After intervention: 82.63 ± 10.30, p<0.001  Mental Health:  Before intervention: 70.06 ± 7.76, p=0.323  After intervention: 88.37 ± 9.45, p<0.001  Energy:  Before intervention: 55.32 ± 6.21, p=0.343  After intervention: 83.53 ± 8.76, p<0.001  Overall Health:  Before intervention: 54.44 ± 6.36, p=0.328  After intervention: 85.33 ± 9.19, p<0.001  **6. Nursing Satisfaction (%)**  Research group: 92.31% satisfied, p = 0.017 | **4. Complication Rates (%)**  Total complication rate: 17.31%  p=0.026  **5. SF-36 Score**  Physiological Function:  Before intervention: 51.85 ± 5.12  After intervention: 72.45 ± 7.46  Physical Pain:  Before intervention: 51.75 ± 7.47  After intervention: 37.68 ± 7.66  Physiological Responsibility:  Before intervention: 73.79 ± 6.56  After intervention: 80.17 ± 7.67  Emotional Responsibility:  Before intervention: 62.45 ± 7.63  After intervention: 75.21 ± 8.33  Social Function:  Before intervention: 52.44 ± 6.53  After intervention: 72.84 ± 8.51  Mental Health:  Before intervention: 71.59 ± 7.88  After intervention: 80.86 ± 8.23  Energy:  Before intervention: 56.48 ± 6.56  After intervention: 76.41 ± 7.24  Overall Health:  Before intervention: 55.59 ± 6.47  After intervention: 76.49 ± 7.14  **6. Nursing Satisfaction (%)**  Control group: 75.00% satisfied |
| Iizuka et al. 2005 | 4 weeks post-operative  8 weeks post-operative | (1) 8-week Cervical Collar group  (2) 4-week Cervical Collar group | **JOA Score**  Group 8W (collar for 8 weeks):  Pre-op: 11.0 ± 2.3  Final Follow-up: 14.5 ± 1.3  Recovery Rate: 55.5 ± 21.7%  Group 4W (collar for 4 weeks):  Pre-op: 10.6 ± 2.8  Final Follow-up: 14.2 ± 2.3  Recovery Rate: 59.0 ± 29.9%  No significant difference in neurologic recovery between the two groups (p > 0.47).  **Cervical ROM and Alignment**  1. Cervical Alignment (°)  Group 8W: Pre-op: 12.6°, Post-op: 12.0°  Group 4W: Pre-op: 13.9°, Post-op: 13.3°  No significant difference between groups in cervical alignment maintenance.  2. Cervical Flexion Range (°)  Group 8W: Pre-op: 30.8°, Post-op: 18.8° (61% retention, p < 0.001).  Group 4W: Pre-op: 34.1°, Post-op: 25.5° (75% retention, p < 0.02).  Group 4W had significantly better maintenance of flexion range than Group 8W.  3. Cervical Extension Range (°)  Group 8W: Pre-op: 13.6°, Post-op: 5.6° (41% retention, p < 0.001).  Group 4W: Pre-op: 12.9°, Post-op: 10.6° (82% retention, p > 0.24).  Extension range was significantly better maintained in Group 4W.  **4. Total Cervical ROM (Flexion + Extension, °)**  Group 8W: Pre-op: 44.5°, Post-op: 24.2° (54% retention).  Group 4W: Pre-op: 47.0°, Post-op: 35.3° (75% retention, p < 0.03).  Total ROM was significantly better maintained in Group 4W. |  |
| mJOA: Modified Japanese Orthopaedic Association; SF-36 PCS: Short Form 36-Item Physical Component Summary; NDI: Neck Disability Index; AMS: American Spinal Injury Association Motor Score; SCIM-III: Spinal Cord Independence Measure Third Version; SCIM-95: Spinal Cord Independence Measure 95th Percentile; SCI-ARMI: Spinal Cord Injury Ability Realization Measurement Index; VAS: Visual Analog Scale; ANOVA: Analysis of Variance; ROM: Range of motion; PT, physiotherapy; OT, occupational therapy; JOA: Japanese Orthopedic Association; EQ-5D-5L: EuroQoL 5-Dimension 5-Level; JOACMEQ: Japanese Orthopedic Association Cervical Myelopathy Evaluation Questionnaire; SVA, sagittal vertical axis; QoL, quality of life; Pre-Int, pre-intervention; SER: Self-Efficacy Assessment for Rehabilitation Outcome; rTMS: Repetitive Transcranial Magnetic Stimulation; RTMS: Right Total Motor Score; LTMS: Left Total Motor Score; UEMS: Upper Extremity Motor Score; LEMS: Lower Extremity Motor Score; JOACMEQ-LEF: Japanese Orthopaedic Association Cervical Myelopathy Evaluation Questionnaire - Lower Extremity Function; TUG, timed up and go, SF-36: Short Form Survey 36-Item Score; UEMS: Upper Extremity Motor Score; LEMS: Lower Extremity Motor Score; JOACMEQ-LEF: Japanese Orthopaedic Association Cervical Myelopathy Evaluation Questionnaire - Lower Extremity Function. | | | | |
